# New Protic Ionic Liquids as Potential Additives to Lubricate Si-Based MEMS/NEMS

**DOI:** 10.3390/molecules28062678

**Published:** 2023-03-16

**Authors:** Mariana T. Donato, Jonas Deuermeier, Rogério Colaço, Luis C. Branco, Benilde Saramago

**Affiliations:** 1 Centro de Química Estrutural, Institute of Molecular Sciences, Departamento de Engenharia Química, Instituto Superior Técnico, Universidade de Lisboa, Av. Rovisco Pais, 1049-001 Lisboa, Portugal; 2 LAQV-REQUIMTE, Departamento de Química, NOVA School of Science and Technology, Universidade NOVA de Lisboa, Campus da Caparica, 2829-516 Caparica, Portugal; 3 CENIMAT|i3N and CEMOP/UNINOVA, Departamento de Ciência dos Materiais, Faculdade de Ciências e Tecnologia, Universidade Nova de Lisboa, Campus da Caparica, 2829-516 Caparica, Portugal; 4 IDMEC-Instituto de Engenharia Mecânica, Departamento de Engenharia Mecânica, Instituto Superior Técnico, Universidade de Lisboa, Av. Rovisco Pais, 1049-001 Lisboa, Portugal

**Keywords:** protic ionic liquids, lubricant additives, friction, wear, silicon, MEMS/NEMS

## Abstract

The motivation for this work was to develop new protic ionic liquids (PILs) as additives for the lubrication of micro and nanoelectromechanical systems (MEMS and NEMS). Ten PILs based on the combination of methylimidazolium ([MIMH]), 4-picolinium ([4-picH]), pyridinium ([PyrH]), 1,8-diazabicyclo[5.4.0]-undec-7-ene-8-ium ([DBUH]) and tetramethylguanidinium ([TMGH]) cations with hydrogen sulfate([HSO_4_]) and mesylate ([MeSO_3_]) anions were tested as additives in polyethylene glycol (PEG200) to lubricate steel/silicon and silicon/silicon contacts. The best additive was [4-picH][HSO_4_], which adsorbed strongly on the Si surface, leading to a protective film that reduced wear by up to 15 times compared to PEG200.

## 1. Introduction

Protic ionic liquids (PILs) are a subclass of ionic liquids, which are composed of Brønsted acids and bases. The unique properties of these PILs result from the presence of proton-donor and proton-acceptor sites, which are responsible for the formation of dense hydrogen bonding leading to bi-continuous sponge-like nanostructures [1]. Greaves and Drummond first reviewed the properties and applications of PILs in 2008 [2] and reported only one study on the lubricant properties of a series of alkylammonium PILs [3]. The authors of that study compared the tribological properties of PILs and imidazolium aprotic ILs with those of mineral oils and found reduced friction and wear of steel-aluminum contacts with the PILs, in particular, with the monoprotic trioctylammonium bis(trifluoromethylsulfonyl)imide ([N_8,8,8,H_][NTf_2_]). This PIL added to a commercial mineral oil eliminated the known adhesion problems in the aluminum sliding surfaces, which was attributed to the fast reaction of the IL with aluminum leading to a protective boundary film. Since then, several authors have been studying the application of ammonium and phosphonium-based PILs as neat lubricants or additives to lubricate metallic contacts [4,5,6,7,8,9,10,11,12,13]. Ammonium-based PILs demonstrated excellent performance as lubricants of magnetic thin films [4], as additives in lithium complex grease to lubricate steel-steel pairs [5] and both as pure lubricants or oil additives in the lubrication of copper-copper contacts [6]. Ultra-low friction was obtained with water containing 2-hydroxyethylammonium succinate to lubricate sapphire-stainless steel contacts [7]. 2-Hydroxyethylammonium formiate and pentanoate were tested as lubricants of aluminum-steel contacts and were demonstrated to be effective in wear reduction, although they did not improve friction in comparison with commercial oils [8]. Similar behavior was observed with other ammonium-based PILs containing the oleate cation: tribological tests with alumina/aluminum pair demonstrated that the only advantages compared to the commercial lubricant were the low price and low toxicity as well as the higher chemical stability [9]. The PIL tri-[bis(2-hydroxyethyl)ammonium] citrate was successfully applied as an additive to mineral oil in the lubrication of aluminum-steel [10] and steel-steel contacts [11], while di[bis(2-hydroxyethyl)ammonium] succinate was used as an additive in water and as base lubricant for graphene dispersions to lubricate alumina/steel pairs [12]. Kahn et al. synthesized fatty-acid-derived PILs based on the phosphonium cation, which decreased friction and wear of the steel tribopair when added to lube oil [13]. It is also important to emphasize that PILs are easily prepared by simple protonation of the cation using appropriate acid, as well as cheaper compared to other ILs.

To our knowledge, the capacity of PILs to lubricate silicon surfaces was never reported in the literature, except in a recent publication by our group [14], as described later in this section. The high demand for the efficient lubrication of micro and nanoelectromechanical systems (MEMS and NEMS), which are traditionally made of silicon, derives from the fact that silicon is a very fragile material and non-adequate lubrication may lead to adhesion, friction and wear problems [15]. These problems occur only when MEMS and NEMS have moving parts, such as in optical switches, magnetic storage devices, resonators, gyroscopes and micro pumps, among others. The peculiar properties of ILs, including their high electrical conductivity which ensures low contact resistance between sliding parts, represent important advantages compared to conventional lubricants in this type of application. Even used as lubricant additives, ILs were found to increase the conductivity of the solvent [16,17].

The investigation of the performance of ILs as neat lubricants or additives in the lubrication of silicon dates back to 2006 when Yu et al. demonstrated that films of vinyl groups functionalized ILs reduced the friction and wear of hydroxyl-terminated Si surfaces [18]. Bhushan et al. [19] evaluated the nanoscale tribological performance of two imidazolium-based ILs deposited on Si wafers. The partially bonded coatings demonstrated the best tribological behavior as they combined the bonded lubricant with a mobile fraction. Zhu et al. [20] and Mo et al. [21,22] investigated the lubrication capacity of a thin film of ILs deposited on single crystal Si wafers. They found that the nano and microtribological properties were dependent on the structure of both the anions and the cations, wettability and environment. Pu et al. [23] used the same type of approach with crown-type phosphate ILs to lubricate silicon surfaces modified with self-assembled monolayers. The group of Spencer [24] studied the lubrication of the silicon/silica pair by two ILs based on the 1-ethyl- and 1-hexyl-3-methylimidazolium cations combined with tris(pentafluoroethyl) tris(perfluoroalkyl)trifluorophosphate anions, respectively, [C_2_MIM][FAP] and [C_6_MIM][FAP], with a pin-on-disk tribometer. They found that varying the environmental humidity affected the mechanism of lubrication in different ways depending on the applied load. Later, they used 1-ethyl-3-methylimidazolium ethylsulfate ([C_2_MIM][EtSO_4_]) in similar studies and claimed that the presence of water in the IL induced decreases in wear and roughness of the sliding surfaces, minimizing the formation of debris [25]. In 2017, the same authors reported a comparison of the lubrication of the silicon/silica tribopair with 1-ethyl-, 1-hexyl- and 1-dodecyl-3-methylimidazolium bis(trifluoromethylsulfonyl)imide, ([C_2_MIM][NTf_2_], [C_6_MIM][NTf_2_] and [C_12_MIM][NTf_2_]), under boundary conditions, with the results obtained using [C_2_MIM][EtSO_4_] and the FAP-based ILs [26]. Raman and XPS spectra of the worn surfaces revealed mechanical wear as the prevailing form when the lubricants were the ILs based on the [EtSO_4_] and the [NTf_2_] anions but not with the ILs containing the [FAP] anion. Our group investigated the use of ILs as additives in the model lubricant polyethylene glycol (PEG200) to lubricate the pair steel ball/Si surface [14,27,28]. A series of imidazolium-based ILs were tested and a significant reduction in the friction coefficient (CoF) was obtained with PEG200 + 2% [C_2_MIM][EtSO_4_], which was attributed to the strong interaction of the anion with the Si surface [27]. Picolinium-based ILs, namely 1-hexyl-2, 3 or 4-picolinium trifluoromethanesulfonate, were also tested in similar studies and [C_6_-4-pic][TfO] demonstrated the best tribological performance which was explained by the ordered adsorbed layer formed by the symmetric cation [28]. Sulfur-based ILs and organic salts, some liquids and other solids at room temperature, were used as additives in the base oil PEG200 [14]. Excellent tribological properties were achieved with ILs containing the methylsulfate ([MeSO_4_]) and the mesylate ([MeSO_3_]) anions. This performance was attributed to the formation of chemical bonds between the S atoms of the anions and the Si surface leading to the formation of a compact adsorbed film. The behavior of aprotic ILs was compared to two PILs such as methylimidazolium mesylate ([MIMH][MeSO_3_]) and tetramethylguanidinium mesylate ([TMGH][MeSO_3_]). The PILs outperformed the aprotic ILs in the reduction in friction compared to the model lubricant (PEG200), except in the case of 1-methyl-3-picolinium methylsulfate ([C_1_-3-pic][MeSO_4_]). However, more extensive studies are needed to confirm the promising results obtained with the investigated PILs.

**Figure 1:**
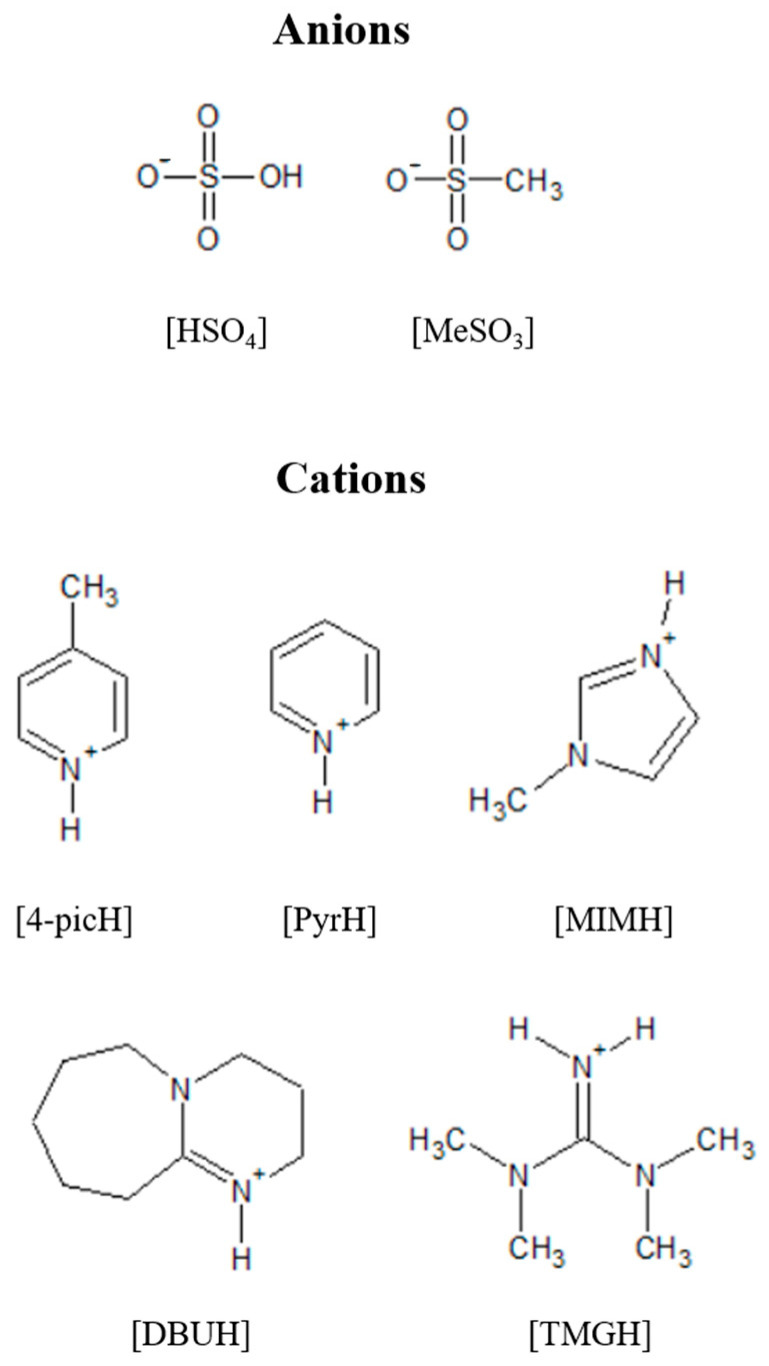
Molecular structures of the studied anions and cations.

In this work, we synthesized ten PILs based on the combination of the methylimidazolium ([MIMH]), 4-picolinium ([4-picH]), pyridinium ([PyrH]), 1,8-diazabicyclo[5.4.0]-undec-7-ene-8-ium ([DBUH]) and tetramethylguanidinium ([TMGH]) cations with hydrogen sulfate ([HSO_4_]) and [MeSO_3_] anions. The molecular structures of these ions are presented in Figure 1. Among these salts, [4-picH][MeSO_3_], [PyrH][HSO_4_], [DBUH][HSO_4_] and [MIMH][HSO_4_], are liquid and the remaining ones are solids at room temperature. The idea was to ally the good lubrication capacity of the anions based on sulfur units with the presence of proton donors in the cations. The PILs were tested as additives to PEG200 in the proportion of 2% in weight. The viscosity and wettability of the mixtures were measured and their lubrication capacity was assessed using two tribopairs: steel sphere/Si surface and Si sphere/Si surface. A first screening of the additives was conducted with the steel spheres, and then the more expensive Si spheres were used to compare the behavior of the worst and the best-performing liquids in the lubrication of Si contacts that better mimic dynamic MEMS/NEMS, most frequently made of silicon. Friction and wear were determined and the worn sliding surfaces were imaged and chemically analyzed in order to understand the wear mechanisms.

## 2. Results and Discussion

### 2.1. Characterization of the Mixtures

The choice of the optimal concentration of the PIL was based on the comparison of the CoF obtained with mixtures of [4-picH][HSO_4_] and PEG200 using three concentrations: 1%, 2% and 5% (*w/w*) (Figure S1). The CoF decreased when the IL concentration increased from 1% to 2% and then increased to 5%. A possible explanation is that the concentration of 1% was not enough to ensure the formation of a protective lubrication film, while 5% led to a rougher film. Thus, a concentration of 2% was adopted for all PILs.

Rheological studies were made at 25 °C and variable shear rate, which showed that the liquid mixtures present a Newtonian behavior. The water content, the viscosity at 25 °C and the contact angle on Si of PEG200 and of the mixtures PEG 200 + 2% PILs are given in Table 1. All mixtures present moderate viscosity, varying between 49 mPa∙s and 64 mPa∙s, higher than pure PEG200. The additives based on the anion [MeSO_3_] lead to higher viscosity than those with the anion [HSO_4_] when sharing the same cation. The PEG200 and all the mixtures exhibited good wettability on the Si surface.

### 2.2. Tribological Tests with the Pair SS/Si

**Figure 2:**
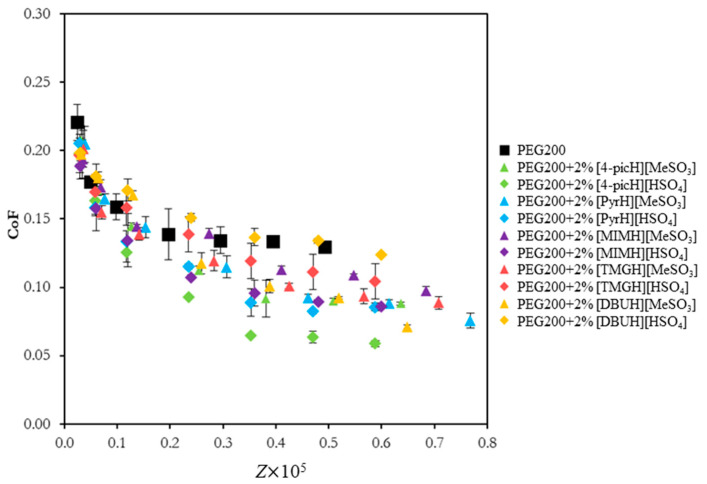
CoF vs. Sommerfeld parameter, Z, for the pair SS sphere/Si substrate using neat PEG200 and the mixtures PEG200 + 2% PIL as lubricants. The errors are ±standard deviation (n ≥ 3).

The first experiments aimed at a previous evaluation of the lubrication capacity of the prepared PILs using the tribopair SS sphere/Si substrate in short tests under a low load (1 N). These conditions were chosen in order to avoid wear, which complicates the interpretation of the friction results, thus allowing a first screening of the PILs. The Stribeck curves (CoF vs. Sommerfeld parameter, Z) for the two sets of PILs sharing the same anions, [HSO_4_] and [MeSO_3_], are shown in Figure 2. The parameter Z is defined as:

(1) $$Z=\frac{ƞvr}{F}$$

where η is the lubricant viscosity (Pa·s), v is the sliding speed (m·s^−1^), r is the counter-body radius (m) and F is the applied load (N). Stribeck curves allow for the identification of the lubrication regimes that exist between contacting surfaces. In this case, two different regimes can be identified: boundary regime, in which the asperities of the surfaces in relative motion come into physical contact and adhesion and/or abrasion occur; boundary/mixed regime, which is an intermediate regime where there is some contact between asperities but the lubricant film already plays an important role in supporting the applied load. In the first regime (Z × 10^5^ < 0.3), CoF decreases sharply with speed, while in the second one (Z × 10^5^ > 0.3), CoF is almost constant.

Apart from [DBUH][HSO_4_], the addition of the PILs to PEG200 led to a decrease in CoF. Most PILs present a similar behavior, but [4-picH][HSO_4_] stands out as the best one. Figure S2A,B compare the Stribeck curves obtained for the PILs grouped according to the anion, [HSO_4_] and [MeSO_3_], respectively. Among the PILs based on the same anion, it is possible to identify significant differences, which seems to indicate that the cation possesses a more important role in determining the lubrication capacity. The small, symmetric cation [4-picH] may interact with the Si surface through hydrogen bonds established between NH and CH groups of the picolinium ring and the oxidized Si, leading to the formation of a compact adsorbed layer. A similar behavior was observed by the authors with 1-methyl-3-picolium methylsulfate ([C_1_-3-pic][MeSO_4_]) [14]. In contrast, the cation [DBUH] has a large size, low symmetry and stronger delocalized charge [29], which do not favor the interaction with the Si surface. The [HSO_4_] anion may also contribute to the formation of the stable lubrication film through the interaction of the S-O groups with the non-oxidized Si, while the hydroxyl groups interact with the Si-O. Thus, the best tribological behavior obtained with [4-picH][HSO_4_] should result from the synergy of the two ions.

We must stress that the tribological behavior of the mixtures PEG 200 + 2% [MIMH][MeSO_3_] and PEG 200 + 2% [TMGH][MeSO_3_] was previously tested with the same tribopair under different conditions (loads of 15 mN and 30 mN) [14]. They led to significant decreases in CoF when compared to PEG200 and to other mixtures of PEG200 with aprotic ILs. However, Figure 2 shows that the tribological behavior of the former mixtures under 1 N of load was clearly outperformed by PEG 200 + 2% [4-picH][HSO_4_]. The additive [4-picH][HSO_4_] reduced 55% the CoF of PEG200 (v = 20 mm·s^−1^), while the reductions with [MIMH][MeSO_3_] and [TMGH][MeSO_3_] were 25% and 31%, respectively.

### 2.3. Tribological Tests with the Pair Si/Si

**Figure 3:**
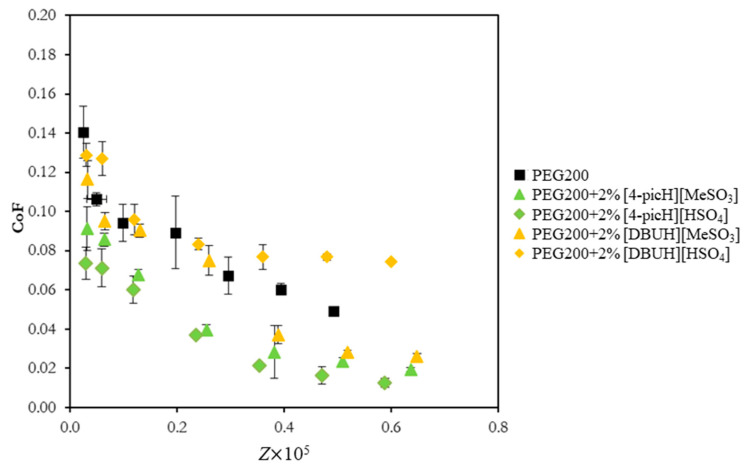
CoF vs. Sommerfeld parameter, Z, for the pair Si sphere/Si substrate using neat PEG200 and the mixtures PEG200 + 2% PIL as lubricants in short tests under load of 1 N. The errors are ±standard deviation (n ≥ 3).

The best and the worst additives tested with the tribological pair SS/Si, respectively, [4-picH][HSO_4_] and [DBUH][HSO_4_], were further studied using the tribopair Si sphere/Si substrate. To better assess the role of the anion, the results are compared with those obtained with the two other PILs sharing the same cations but with the anion [MeSO_3_]: [4-picH][MeSO_3_] and [DBUH][MeSO_3_]. Figure 3 shows CoF vs. Z for neat PEG200 and the mixtures of these additives with PEG200 obtained with short tests under the load of 1 N. When these results are compared with the previous ones obtained with the tribopair SS/Si, a significant reduction in the CoF is observed for all mixtures. This decrease may be attributed to the smaller roughness of the Si spheres (RMS = 15 nm) compared to the SS spheres (RMS = 1 µm).

The tribological behavior of the tested PILs follows a similar pattern to that obtained with the SS spheres but with a slight difference: [DBUH][HSO_4_] is still the worst additive but the lubrication capacities of [4-picH][HSO_4_] and [4-picH][MeSO_3_] are almost the same. A possible justification for this behavior should lie in different interactions between these anions and the SS or Si spheres. It seems that the anion [HSO_4_] may react with steel to produce a protective layer more efficiently than [MeSO_3_], thus justifying the better behavior of [4-picH][HSO_4_] for the SS/Si pair.

**Figure 4:**
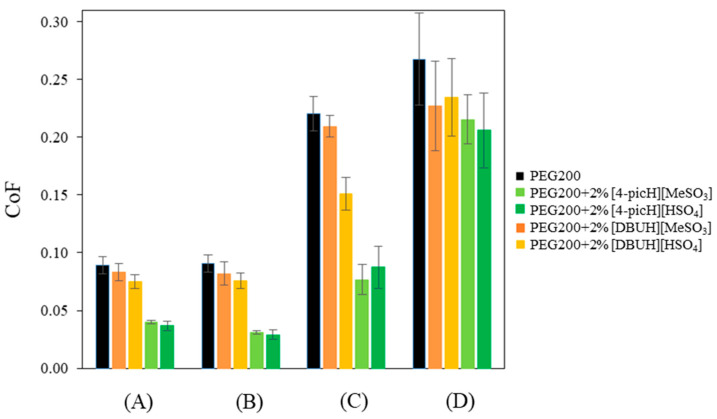
Average CoF values obtained with the pair Si sphere/Si substrate using neat PEG200 and the mixtures PEG200 + 2% PIL as lubricants: short tests under the load of 1 N (A) and long tests under the loads of 1 N (B), 2 N (C) and 4 N (D). The errors are ±standard deviation (n ≥ 3).

The influence of the load and the number of cycles on the CoF was investigated and the results obtained with forces of 1 N, 2 N and 4 N, at the sliding speed of 8 mm·s^−1^ are presented in Figure 4. The choice of this value was based on the analysis of the Stribeck curves (Figure 3), which show that for this intermediate speed, the lubrication regime may be considered boundary/mixed (low Sommerfeld parameter). To confirm the lubrication regime, the value of the thickness of the lubricant film should be compared with the composite surface roughness, defined as ${\left(\sigma_{ball}^{2}+\sigma_{disk}^{2}\right)}^{1/2}$, which in the present case is approximately 15 nm. Although we do not have direct experimental access to that thickness, we may have an estimation through the AFM analysis of the film that remained on the Si surface, following the tribological experiments. Figure S3 shows the surface of the Si substrate at the end of the test with PEG200 + 2% [4-picH][HSO_4_] (2375 cycles, 1 N) after removing the excess liquid: outside the track an adsorbed layer approximately 4.5 nm high is visible. The observed track should be the result of the continuous removal of this layer during the sliding movement of the tribological pair. Furthermore, we estimated the theoretical minimum film thickness by applying the elastohydrodynamic theory of lubrication (EHL) to a nonconformal geometry of ball-on-disk contact [30]. The value of the film thickness obtained for the load of 1 N (condition of Figure 3) and sliding speed of 8 mm·s^−1^ (details of the calculation in Supporting Information) is ~2 nm. We must stress that the calculated value of the film thickness must be taken with care, because the EHL theory of lubrication does not strictly apply when the film thickness is in the order of nanometers, and inconsistencies were found when applying the EHL theory to ILs [31]. Nevertheless, the consistency between the calculated minimum film thickness and the thickness measured with AFM allows concluding that the film is not thick enough to separate the two sliding surfaces, confirming a boundary/mixed lubrication regime, where the load is carried mainly by the surface asperities or partially by the asperities and the lubricant film.

**Figure 5:**
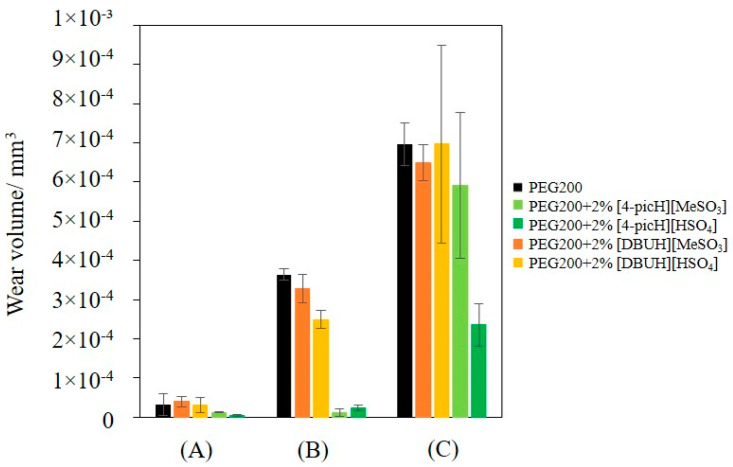
Average wear volumes obtained with the pair Si sphere/Si substrate using neat PEG200 and the mixtures PEG200 + 2% PIL as lubricants in long tests under loads of 1 N (A), 2 N (B) and 4 N (C). The errors are ±standard deviation (n ≥ 3).

At low load, the value of the CoF is constant throughout all the sliding distances of the tribological test: the average values obtained with 2375 cycles are not significantly different from those obtained with 85 cycles. In contrast, increasing the load led to increasing values of CoF, with a larger scatter, indicating the existence of wear. In all cases, the presence of the additives decreased the average CoF, but the larger decreases occurred with [4-picH][HSO_4_] and [4-picH][MeSO_3_]. As expected, after the short tests, no wear of the Si substrates could be detected, but the situation changed for the longer tests. Figure 5 shows the wear volumes obtained in long tests under loads of 1 N, 2 N and 4 N.

**Figure 6:**
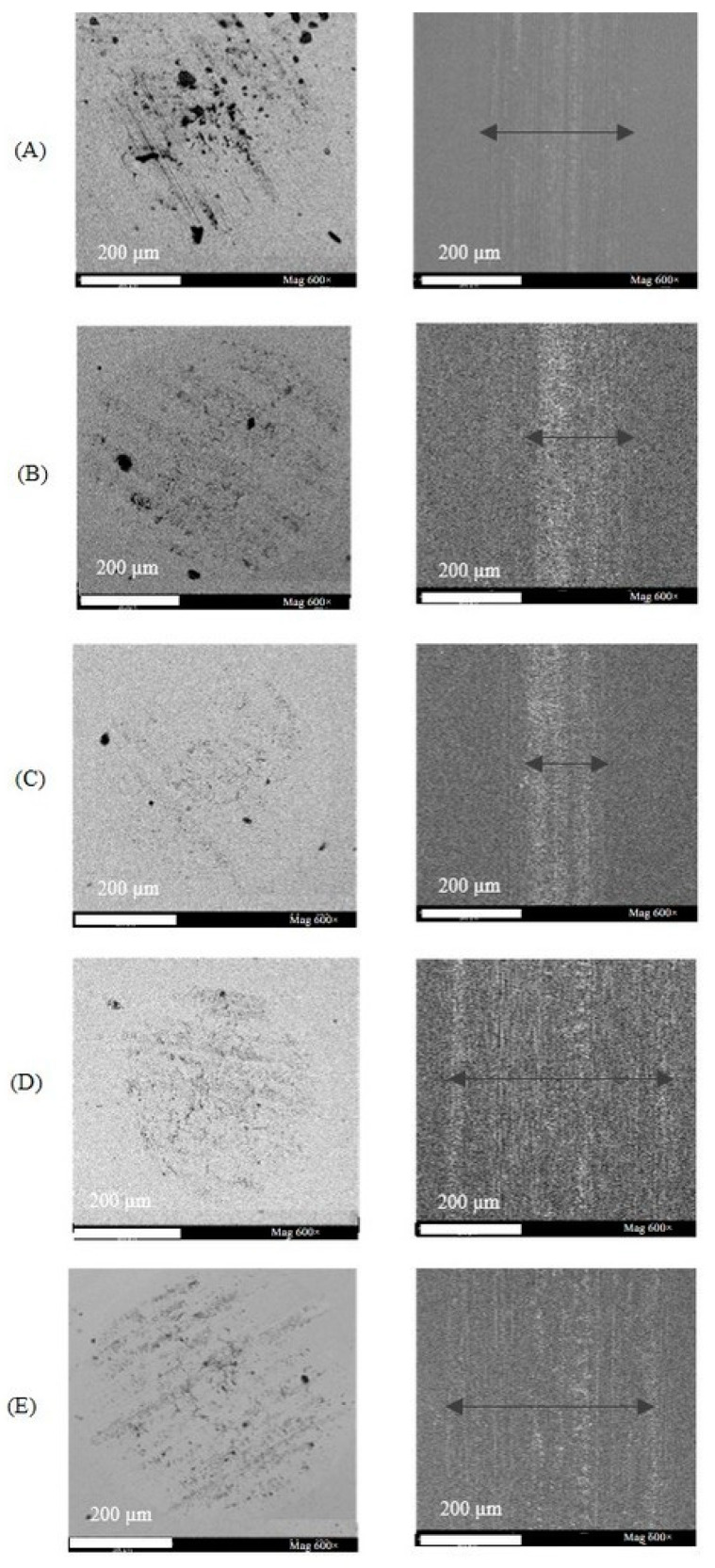
FEG-SEM images with 600× amplification of the worn surfaces of the Si balls and the Si substrates after long tribological tests under the load of 4 N using as lubricants: PEG200 (**A**), PEG200 + 2% [4-picH][MeSO_3_] (**B**), PEG200 + 2% [4-picH][HSO_4_] (**C**), PEG200 + 2% [DBUH][MeSO_3_] (**D**) and PEG200 + 2% [DBUH][HSO_4_] (**E**). The black arrows mark the limits of the wear tracks.

The most remarkable results were obtained with the additives [4-picH][HSO_4_] and [4-picH][MeSO_3_] at 2 N: the wear volumes became almost negligible. For the lower load (1 N), the wear volumes were small for all mixtures, while for the higher load (4 N), the large scatter of the results does not allow a meaningful comparison, although [4-picH][HSO_4_] still demonstrated the best behavior. The analysis of the time dependence of the CoF in the long tests helps to understand the dispersion of the CoF and the wear values. Figure S4 presents examples of the variation with time of CoF obtained during long tests with v = 8 mm·s^−1^ for 1 N, 2 N and 4 N. The CoF values remained constant without the presence of any running-in period for the tests under 1 N, indicating the stability of the lubricant film. Under the load of 2 N, only the test with PEG200 + 2% [4-picH][HSO_4_] led to a constant value, although the variation was very slight with PEG200 + 2% [4-picH][MeSO_3_]. The tests under 4 N led to some instability with sporadic peaks in the CoF values for all mixtures giving evidence to the existence of three-body wear. The CoF is slightly decreasing with time, which may be attributed to the rolling and sliding of debris particles that absorb deformation energy [32]. The same behavior can be detected in the tests under 2 N using PEG200 and their mixtures with [DBUH][HSO_4_] and [DBUH][MeSO_3_]. Silicon is known to have good mechanical properties but its low toughness results in adhesive wear where the first debris are formed, followed by third-body wear [33,34]. The local variations of the surface morphology should be responsible for the observed fluctuations in the friction coefficient. The additives [4-picH][HSO_4_] and [4-picH][MeSO_3_] strongly adsorb on the Si surfaces of the body and the counter-body minimizing the contact between the sliding surfaces during the tribological tests under loads of 1 N and 2 N. For the highest load (4 N), the additives could no longer protect the surfaces from contact and third-body wear occurs, as shown in the SEM images in Figure 6. The images of the worn surface of the Si balls clearly show signs of three-body abrasive wear where the black dots correspond to debris. These small black particles of angular shape result from the oxidation of Si fragments detached during the first cycles of the tribological tests and oxidized by the action of heat and pressure. It is clear from the figure that the three-body abrasive wear is more intense for PEG200 and has the lowest intensity when using the additive [4-picH][HSO_4_]. The width of the wear tracks on the Si substrates confirms these results: the narrowest track is observed for [4-picH][HSO_4_].

**Figure 7:**
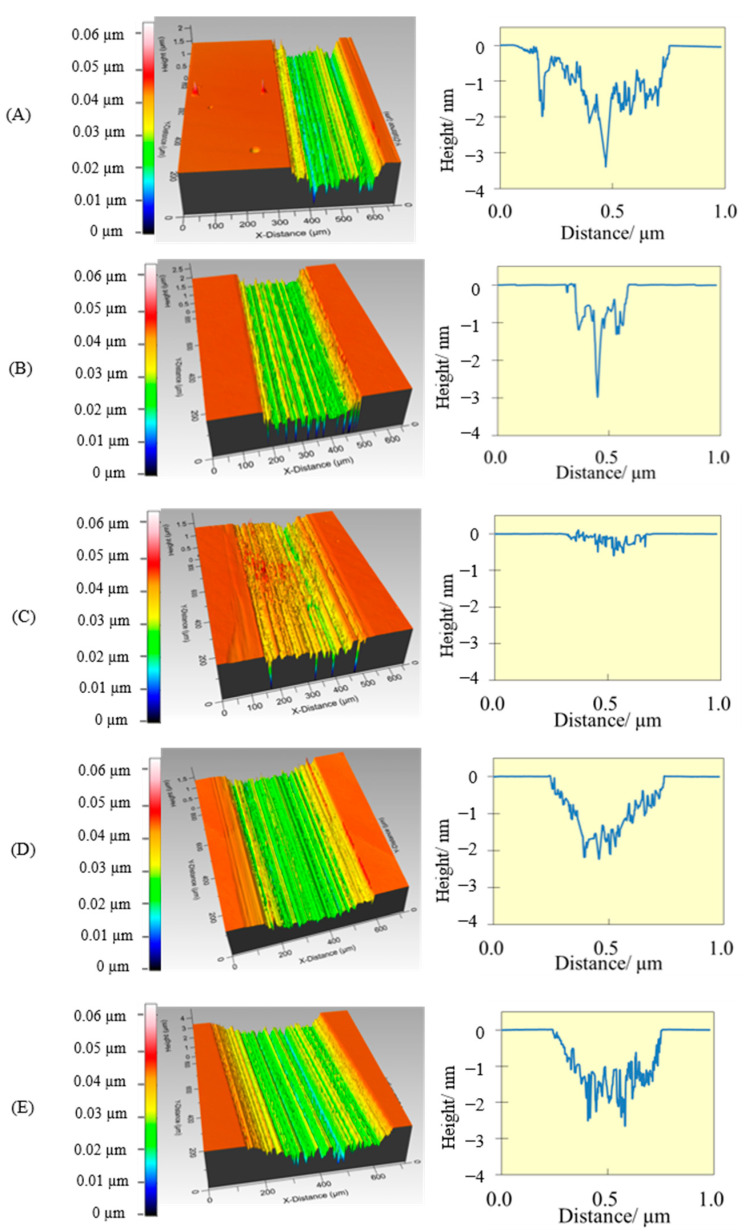
Wear tracks of the Si substrates (profilometer images with 20× amplification) after long tribological tests under the load of 4 N using as lubricants: PEG200 (**A**), PEG200 + 2% [4-picH][MeSO3] (**B**), PEG200 + 2% [4-picH][HSO4] (**C**), PEG200 + 2% [DBUH][MeSO3] (**D**) and PEG200 + 2% [DBUH][HSO4] (**E**).

The profiles of the wear tracks on the Si substrates were obtained with a profilometer and are presented in Figure 7. PEG200 led to a deep, wide wear profile, while the profile obtained with [4-picH][HSO_4_] is the shallowest. This behavior is typical of well-lubricated surfaces of the same material moving relative to each other. The moving surfaces deform and the ball penetrates slightly on the underlying substrate leading to a wide, shallow wear track.

The elemental composition of the Si substrates inside and outside the wear tracks (under the load of 4 N) obtained with XPS analysis is presented in Table 2. The most striking result is the presence of nitrogen inside and outside the track for PEG200 + 2% [4-picH][HSO_4_], the best lubricating mixture. This confirms that [4-picH][HSO_4_] is the PIL with a higher tendency to adsorb on the Si surface. Surprisingly, no sign of sulfur could be detected indicating that the interaction of this PIL with the surface occurs mostly through the cation. When comparing the atomic percentages inside the wear tracks, the percentages of Si are higher and those of oxygen and carbon are lower for the worst additives, which are the ones based on the [DBUH] cation. This is in agreement with the fact that the films of adsorbed [DBUH][MeSO_3_] and [DBUH][HSO_4_] are less resistant to wear and are removed more easily from the surface during the sliding process. Comparison of the Si 2p percentages inside and outside the wear tracks reveals slightly lower values inside, with the exception of the [DBUH][HSO_4_], which suggests that the sliding process helps in the spreading of the lubrication film on the Si substrate. In the case of O 1s and C 1s, the data are difficult to interpret because these elements may derive from different sources, including ambient contamination.

**Figure 8:**
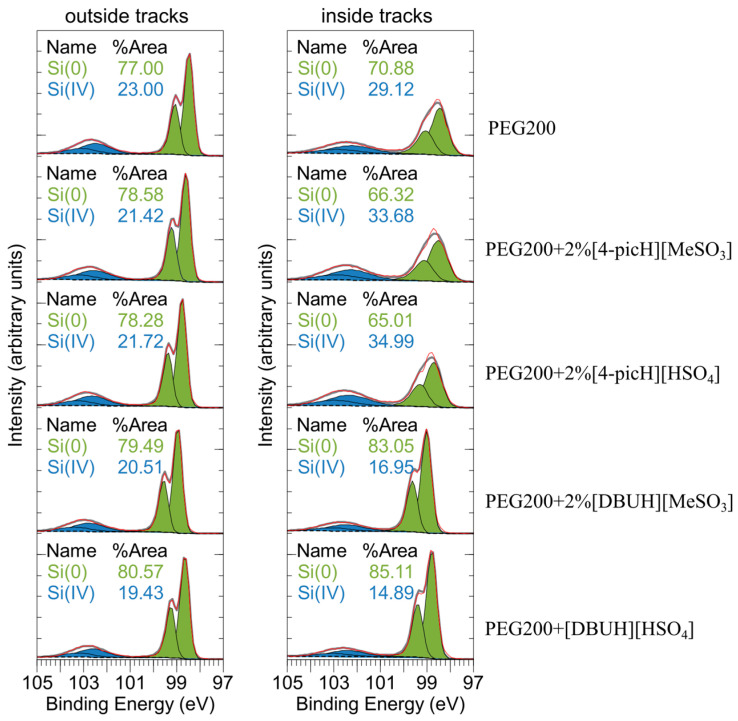
XPS Si 2p emission from outside (**left**) and inside (**right**) of the wear tracks (shown in Figure 6). Red: original data, dashed black line: background, green: Si^0^ components, blue: Si^4+^ components, grey: envelope. Each silicon oxidation state is fitted with two components of the same color, corresponding to the Si 2p_3/2_ and Si 2p_1/2_ doublets, separated by 0.6 eV and with identical line width, respectively.

The XPS spectra of the Si substrates inside the wear tracks (regions limited by the segments between arrows in Figure 6) are compared with the ones outside the wear tracks, in Figure 8. The relative amount of Si^4+^ compared to Si^0^ provides an indication for surface oxidation of the silicon. The quantification was conducted via fitting over a Shirley background, using Si 2p duplets with a line shape of LA (1.5,3,90) for Si^0^ and GL(30) for Si^4+^. The Si substrates outside the wear tracks all have a similar Si^4+^ contribution. After application of the load, the presence of oxidized silicon is most pronounced with the lubricants containing [4-picH]. This strongly indicates the wear-induced formation of silicon dioxide, which offers additional protection against wear. In fact, the Young modulus of SiO_2_ (66–75 MPa) is much smaller than that of Si (130–169 MPa), which makes the oxidized silicon easier to deform. Note, that the superior resistance to wear not only depends on the surface oxidation by the cation [4-picH] but also on the right anion: the combination [4-picH][HSO_4_] allows for better protection of the sliding surfaces, which may be related to its higher chemical adsorption.

**Figure 9:**
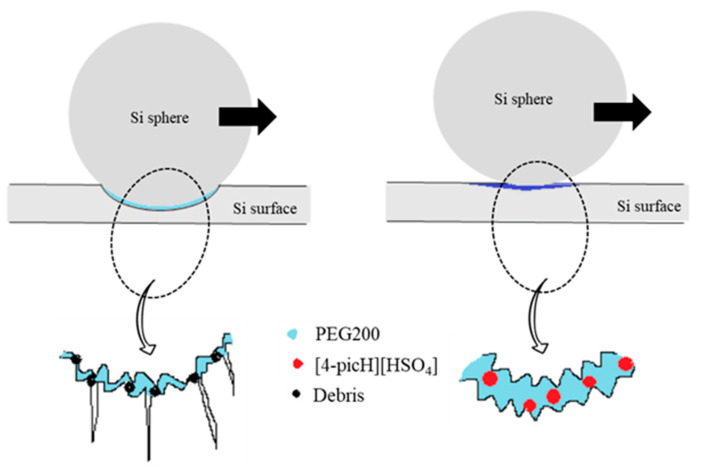
Schematic representation of the lubrication process during the tribological tests under high load.

A schematic representation of the possible lubrication process during the tribological tests under high load with PEG200 and the mixture PEG200 + 2% [4-picH][HSO_4_] is proposed in Figure 9. The three-body abrasive wear leads to the formation of debris and surface cracks, which occurs when the lubricant PEG200 is minimized in the presence of the mixture. The PIL adsorbs to the sliding surfaces avoiding direct contact between the body and counterbody which becomes slightly deformed under the load.

## 3. Materials and Methods

All reagents for the synthesis of the PILs were purchased and used without additional purification. The list of reagents is the following: 4-methylpyridine 98%, methylimidazole 99% and tetramethylguanidine 99% from Alfa Aesar (Tewksbury, MA, USA); pyridine p.a. and sulfuric acid 95–97% from Merk (Rahway, NJ, USA); 1,8-diazabicyclo(5.4.0)undec-7-ene 98% and methanesulfonic acid 99% from Sigma-Aldrich (Rahway, NJ, USA).

The solvents were acetonitrile 99.8% from Merck (Darmstadt, Germany) and deuterated water 99.9% from Eurisotop (Gif sur Yvette, France). Polyethylene glycol (MW 200)—PEG200 was from Sigma-Aldrich (Rahway, NJ, USA), with water content < 0.5%. Distilled and deionized water (DD) was obtained with a Millipore system.

Si b100N wafers (Si-Mat, Kaufering, Germany), with 0.5 mm of thickness, 1 nm of root mean square (RMS) roughness and 1121–1428 HV of hardness, were cut in squares (1 × 1 cm^2^) to be used in the tribological tests and the contact angle measurements. Spheres of stainless steel (SS) AISI 316L grade 100 (Atlas Ball and Bearing Co. Ltd., Walsall, UK) with 6 mm of diameter, roughness RMS = 1 µm and hardness of 260–390 HV and Si spheres (J. Hauser GMBH and Co., Solms, Germany) with 6 mm of diameter, 15 nm of RMS roughness and 1412 HV of hardness were used as counter bodies.

### 3.1. PIL Synthesis

The syntheses of the PILs are described in detail in the Supporting Information. In order to check the chemical structures and purities, all compounds were characterized by ^1^H and ^13^C NMR, FTIR and elemental analysis (see Figure S5 in Supporting Information). The syntheses of [MIMH][MeSO_3_], [TMGH][MeSO_3_] and [DBUH][HSO_4_] were described in previous works [14,35].

### 3.2. Characterization of the Mixtures PIL + PEG200

The choice of the optimal concentration of the PIL was based on the comparison of the CoF obtained with mixtures of [4-picH][HSO_4_] and PEG200 using three concentrations: 1%, 2% and 5% (*w/w*) (Figure S1). The best performance was obtained with 2% (*w/w*), which was adopted for all PILs. The mixtures were dried under a vacuum and their water content was determined by Karl–Fischer coulometric titration (Metrohm, Herisau, Switzerland). The chemical stability of the formulations was checked by ^1^H-NMR comparing the original spectra of the components (PEG200 and PILs) as well as the mixtures PEG200 + 2%PILs after 6 months of preparation. In general, it is possible to conclude that the formulations are chemically stable. In order to prove the chemical stability, ^1^H-NMR of the mixture PEG200 + 2% [4-picH][HSO_4_] was included in the SI (see Figure S6).

The viscosity was measured at 25 °C using a rheometer MCR 92 (Anton Paar, Graz Austria). The results are average values obtained from three measurements. The contact angles of the studied liquids on the Si surface were measured by the sessile drop method at room temperature [36]. The Si substrates were cleaned according to the following protocol: (1) 2 × 15 min sonication in Dextran^®^ solution intercalated with 10 min sonication in water; (2) 3 × 10 min sonication in water; (3) rinsing with DD water. After being flushed with nitrogen, and dried in a vacuum oven at room temperature for, at least two hours, the substrates were placed inside an environmental chamber 100-07-00 (Ramé-Hart, Succasunna, NJ, USA). The images of the sessile drops were captured with a camera jAi CV-A50 (Infaimon, Barcelona, Spain) coupled to a microscope Wild M3Z (Leica Microsystems, Wetzlar, Germany), and the software ADSA (Applied Surface Thermodynamics Research Associates, Toronto, ON, Canada) was used for image analysis. The reported contact angles were average values obtained from measurements performed on at least four drops of each liquid.

### 3.3. Tribological Tests

The tribological tests were conducted with a tribometer TRB3 (Anton Paar, Graz, Switzerland) in the configuration reciprocating ball-on-flat at room temperature (~25 °C) and relative humidity (~45%). The tribopairs were SS spheres/Si substrates and Si spheres/Si substrates. After cleaning both spheres and substrates, using the protocol described in the previous section, the sphere was placed on the tribometer arm and the Si substrate was glued to a metallic container. Several drops of liquid were added to ensure the full coverage of the surfaces. Two types of tests were conducted with the different tribopairs. The SS spheres were used in short-duration tests (85 cycles, corresponding to 0.68 m of sliding distance), under a low normal force of 1 N, and sliding speeds varying between 1 and 20 mm·s^−1^. The Hertz contact stress was 584.5 MPa. The Si spheres were used in short and long tests (2375 cycles, corresponding to 19 m of sliding distance), under the normal load of 1 N and higher normal loads of 2 N and 4 N, at a constant speed of 8 mm·s^−1^. The amplitude of the reciprocal movement of the counter body was always 4 mm. The Hertz contact stresses varied between 584.5 MPa for 1 N and 927.9 MPa for 4 N. The results were analyzed using the software TriboX. The reported values for CoF represent the average of at least three results obtained in independent experiments.

After the tribological tests, the Si spheres and the Si substrates were carefully cleaned with acetone and dried with nitrogen, to remove any traces of adsorbed material. A scanning electron microscope (FEG-SEM) JSM7001F (JEOL, Tokyo, Japan) was used to analyze the surfaces of both Si spheres and Si substrates after the tribological tests. The surfaces of the Si substrates were also imaged using an optical profilometer Profilm 3D (Filmetrics, San Diego, California, USA) and for each track, the worn volume was estimated by multiplying the track length by the average of the cross-sectional areas of the worn track determined by numerical integration of the 2D profiles (3–5 measurements per track).

The elemental composition of the wear tracks on the Si spheres and Si substrates was studied by X-ray photoelectron spectroscopy (XPS), using a spectrometer Axis Supra (Kratos Analytical Ltd., Manchester, UK). A monochromatic Al Kα source was run at 225 W. The detailed spectra were acquired at a pass energy of 20 eV through an aperture of 110 μm. Data analysis was conducted with CasaXPS. Atomic percentages were calculated assuming a homogenous distribution of elements, using the Kratos relative sensitivity factors.

In order to assess the thickness of the film remaining on the Si substrate after the long tribological test at 1 N, the excess liquid was removed with absorbing paper and dried with a nitrogen flow. The tracks left on the adsorbed film were analyzed using an atomic force microscope (NanoSurf Easyscan 2, Liestal, Switzerland) with Si tips (c = 0.2 N·m^−1^, f_0_ = 25 kHz) in contact mode (contact force of 50 nN), using the software WSxM 5.0 Develop 4.0.

## 4. Conclusions

The tribological properties of ten PILs based on the hydrogen sulfate [HSO_4_] and meyslate [MeSO_3_] anions as additives to model lubricant PEG200 were evaluated first in steel/Si and afterward in Si/Si contacts. From the initial screening, two cations were chosen, which led to the best ant the worst lubrication, respectively, [4-picH] and [DBUH]. The lubrication capacity of [4-picH][HSO_4_], [4-picH][MeSO_3_], [DBUH][HSO_4_] and [DBUH][MeSO_3_] was further assessed using Si/Si pairs to mimic the behavior of MEMS and NEMS. The best additive was [4-picH][HSO_4_], which revealed excellent lubrication capacity and minimized third-body abrasive wear. The most striking results were the wear reductions observed at loads of 2 N and 4 N: 15 times and three times lower than that with neat PEG200, respectively. Chemical and image analysis of the wear tracks demonstrated that this PIL, composed of the symmetric cation [4-picH] and the anion [HSO_4_], was able to adsorb strongly on the Si surface leading to the formation of a protective film on the sliding surfaces. We conclude that, through an adequate cation/anion combination, it is possible to prepare PILs that are very promising additives to lubricate efficiently Si-based MEMS/NEMS.

## Figures and Tables

**Table 1:** Water content, viscosity, η, at 25 °C and equilibrium contact angle on Si substrates of PEG200 and the mixtures. The standard deviations correspond to n = 3 for viscosity and n ≥ 4 for contact angle.

Liquids	Water Content/ppm	η/mPa∙s	Contact Angle/°
PEG200	175	41 ± 1	28 ± 2
PEG 200 + 2% [MIMH][HSO_4_]	75	50 ± 1	26 ± 3
PEG 200 + 2% [MIMH][MeSO_3_]	62	56 ± 1	28 ± 2
PEG 200 + 2% [4-picH][HSO_4_]	88	49 ± 1	27 ± 4
PEG 200 + 2% [4-picH][MeSO_3_]	375	53 ± 1	28 ± 4
PEG 200 + 2% [PyrH][HSO_4_]	62	48 ± 1	26 ± 4
PEG 200 + 2% [PyrH][MeSO_3_]	486	63 ± 1	28 ± 4
PEG 200 + 2% [DBUH][HSO_4_]	138	50 ± 1	22 ± 2
PEG 200 + 2% [DBUH][MeSO_3_]	75	54 ± 1	28 ± 4
PEG 200 + 2% [TMGH][HSO_4_]	112	49 ± 1	18 ± 2
PEG 200 + 2% [TMGH][MeSO_3_]	2875	58.7 ± 0.5	27 ± 4

**Table 2:** Relative surface atomic concentrations inside and outside the wear tracks (shown in Figure 6).

Lubricant	Relative Atomic Percentages							
	Si 2p		O 1s		C 1s		N 1s	
	In	Out	In	Out	In	Out	In	Out
PEG200	37.4	45.4	31.8	35.9	30.8	18.7	-	-
PEG200 + 2% [4-picH][MeSO3]	36.4	48.3	32.2	33.3	31.4	18.4	-	-
PEG200 + 2% [4-picH][HSO4]	37.8	41.6	34.1	33.3	27.7	24.6	0.4	0.5
PEG200 + 2% [DBUH][MeSO3]	49.2	52.1	27.7	34.9	23.1	13.0	-	-
PEG200 + 2% [DBUH][HSO4]	48.7	45.5	28.2	21.1	23.1	33.4	-	-

## Data Availability

Data is contained within the article or Supplementary Materials.

## Supplementary Materials

The following information is available online at https://www.mdpi.com/article/10.3390/molecules28062678/s1; Description of the syntheses. Supplementary Figure S1: CoF vs. sliding velocity obtained with three concentrations of [4-PicH][HSO_4_] in PEG200: 0% (■), 1% (■), 2% (▲) and 5% (●). The errors are ±standard deviation (n ≥ 3). Supplementary Figure S2: CoF vs. Sommerfeld parameter, *Z*, for neat PEG200 and the mixtures PEG200 + 2% PIL: (A) PILs based on the anion [HSO_4_]^−^ and (B) PILs based on the anion [MeSO_3_]^−^. The errors are ±standard deviation (n ≥ 3). Supplementary Figure S3: AFM image of the film remaining on the Si substrate after the tribological test with PEG200 + 2% [4-picH][HSO_4_] (2375 cycles, 1 N). The insert represents the magnification of marked area on the track an adsorbed layer. Supplementary Figure S4: CoF as a function of time obtained in long tests with the pair Si/Si, under 1 N, 2 N and 4 N, and v = 8mm·s^−1^ using PEG200 and the mixtures of PEG200 with [4-picH][MeSO_3_], [4-picH][HSO_4_], [DBUH][MeSO_3_] and [DBUH][HSO_4_] as lubricants. Supplementary Figure S5: ^1^H-NMR, ^13^C-NMR and FTIR spectra of the synthesized PILs. Supplementary Figure S6: ^1^H-NMR spectrum of the mixture PEG200 + 2% [4-picH][HSO_4_]. Calculation of the theoretical minimum film thickness using elastohydrodynamic theory of lubrication (EHL) [37,38,39].
