# Supplementary material for: New Protic Ionic Liquids as Potential Additives to Lubricate Si-Based MEMS/NEMS
Source: Molecules. 2023 Mar 16;28(6):2678. doi: 10.3390/molecules28062678 (PMC10057895; doi:10.3390/molecules28062678)
Supplement: Supplementary file 1 [file molecules-28-02678-s001.zip › molecules-2279374-supplementary.pdf]

Supporting Information

New protic ionic liquids as potential additives to lubricate Si-based MEMS/NEMS

Mariana T. Donato [1,2], Jonas Deuermeier [3], Rogério Colaço [4], Luis C. Branco [2,*]
and Benilde Saramago [1,*]

1 Centro de Química Estrutural, Institute of Molecular Sciences, Departamento de Engenharia Química,
Instituto Superior Técnico, Universidade de Lisboa, Av. Rovisco Pais, 1049-001 Lisboa, Portugal
2 LAQV-REQUIMTE, Departamento de Química, NOVA School of Science and Technology, Universidade NOVA de Lisboa, Campus da Caparica, 2829-516 Caparica, Portugal
3 CENIMAT|i3N and CEMOP/UNINOVA, Departamento de Ciência dos Materiais, Faculdade de Ciências e
Tecnologia, Universidade Nova de Lisboa, Campus da Caparica, 2829-516 Caparica, Portugal
4 IDMEC-Instituto de Engenharia Mecânica, Departamento de Engenharia Mecânica, Instituto Superior
Técnico, Universidade de Lisboa, Av. Rovisco Pais, 1049-001 Lisboa, Portugal
* Correspondence: l.branco@fct.unl.pt (L.C.B.); b.saramago@tecnico.ulisboa.pt (B.S.);
Tel.: +351-965895307 (L.C.B.); +351-968280833 (B.S.)

Synthesis

4-picolinium mesylate: [4-picH][MeSO$_3$]

2.1 mL of 4-picoline (0.02147 mol) and 1 equivalent of methanesulfonic acid (1.4 mL) were added to a 50 mL round-bottom flask. A total of 30 mL of acetonitrile was added and the mixture was stirred for 24h at room temperature. The solvent was evaporated and the final product was dried in vacuum and obtained as a white solid (quantitative yield).

^{1}H-NMR (δ, D$_2$O, 400 MHz): 8.61 (d, 2H, J=4.0 Hz), 7.91 (d, 2H, J= 4.0 Hz), 2.81 (s, 3H), 2.68 (s, 3H) ppm.

^{13}C-NMR (δ, D$_2$O, 100 MHz): 161.70, 139.93, 127.84, 38.42, 21.69 ppm.

FTIR-ATR: $\bar{\nu}$ = 3411 (NH), 1641 (C=N), 1508 (CC aromatic), 1162 (C-SO$_2$), 1040 (C-O), 775 (CH aromatic), 517 (CH aromatic) cm^{-1}.

Elemental analysis $C_7H_{11}NO_3S\cdot 1.4H_2O$: expected C 39.20%, H 6.12%, N 6.53%; found C 39.26%, H 6.16%, N 6.49%.

4-picolinium hydrogen sulfate: [4-picH][HSO$_4$]

2.1 mL of 4-picolinium (0.02147 mol) and 1 equivalent of sulfuric acid (1.15 mL) were added to a 50 mL round-bottom flask. 25 mL of acetonitrile were added and the mixture was stirred for 24h at room temperature. The solvent was evaporated and the final product was dried in vacuum and obtained as a white solid (quantitative yield).

^{1}H-NMR (δ, $CDCl_3$, 400 MHz): 14.37 (s, 1H), 8.47 (m, 2H), 7.66 (m, 2H), 2.31 (s, 3H) ppm.

^{13}C-NMR (δ, D_2O, 100 MHz): 161.64, 139.93, 127.81, 21.67 ppm.

FTIR-ATR: $\bar{\nu}$ = 3416 (NH), 3081 (OH), 1640 (C=N), 1507 (CC aromatic), 1156 (C-O), 1029 (C-O), 855 (CH aromatic), 794 (CH aromatic), 571 (CH aromatic), 476 (CC aliphatic) cm^{-1}.

Elemental analysis $C_6H_9NO_4S\cdot1.2H_2O$: expected C 33.86%, H 5.22%, N 6.58%; found C 33.85%, H .15%, N 6.53%.

Pyridinium mesylate: [PyrH][MeSO$_3$]

2 mL of pyridine (0.02528 mol) and 1 equivalent of methanesulfonic acid (1.64 mL) were added to a 100 mL round-bottom flask. A total of 50 mL of acetonitrile was added and the mixture was stirred for 24h at room temperature. The solvent was evaporated and the final product was dried in vacuum and obtained as a white solid (quantitative yield).

^{1}H-NMR (δ, D_2O, 400 MHz): 8.80 (d, 2H, J=4.0 Hz), 8.64 (t, 1H, J= 8.0 Hz), 8.09 (t, 2H, J= 8.0 Hz), 2.79 (s, 3H) ppm.

^{13}C-NMR (δ, D_2O, 100 MHz): 147.19, 141.14, 127.42, 38.43 ppm.

FTIR-ATR: $\bar{\nu}$ = 3674 (OH), 2972 (CH), 2902 (CH), 1621 (C=N), 1548 (CC aromatic), 1491 (C=C-C aromatic), 1145 (C-SO_2), 1021 (C-O), 748 (CH aromatic), 678 (CH aromatic), 608 (CH aromatic), 526 (CH aromatic) cm^{-1}.

Elemental analysis $C_6H_9NO_3S$: expected C 41.13%, H 5.18%, N 7.99%; found C 41.13%, H 5.39%, N 8.03%.

Pyridinium hydrogen sulfate: [PyrH][HSO$_4$]

2 mL of pyridine (0.02528 mol) and 1 equivalent of sulfuric acid (1.36 mL) were added to a 50 mL round-bottom flask. A total of 30 mL of acetonitrile was added and the mixture was stirred for

24h at room temperature. The solvent was evaporated and the final product was dried in vacuum and obtained as an off-white solid (quantitative yield).

^{1}H-NMR (δ, D_2O, 400 MHz): 8.77 (m, 2H), 8.62 (m, 1H), 8.07 (m, 2H) ppm.

^{13}C-NMR (δ, D_2O, 100 MHz): 147.20, 141.07, 127.41 ppm.

FTIR-ATR: $\bar{\nu}$ = 3661 (OH), 2972 (CH), 2900 (CH), 1618 (CC aromatic), 1545 (NH), 1488 (C=C-C aromatic), 1151 (C-O), 1032 (C-O), 842 (CH aromatic), 748 (CH aromatic), 678 (CH aromatic), 526 (CH aromatic) cm^{-1}.

Elemental analysis $C_5H_7NO_4S{\cdot}0.7H_2O$: expected C 31.64%, H 4.79%, N 7.38%; found C 31.74%, H 4.24%, N 7.47%.

Methylimidazolium mesylate: [MIMH][MeSO$_3$]

The synthesis of this PIL was performed according to a previously reported method.

Methylimidazolium hydrogen sulfate: [MIMH][HSO$_4$]

2 mL of methylimidazole (0.02436 mol) and 1 equivalent of sulfuric acid (1.33 mL) were added to a 50 mL round-bottom flask. A total of 35 mL of acetonitrile was added and the mixture was stirred for 24h at room temperature. The solvent was evaporated and the final product was dried in vacuum and obtained as a pale yellow viscous liquid (quantitative yield).

^{1}H-NMR (δ, DMSO, 400 MHz): 9.05 (s, 1H), 7.69 (m, 2H), 6.89 (s, 1H), 3.87 (s, 3H) ppm.

^{13}C-NMR (δ, D_2O, 100 MHz): 134.96, 122.93, 119.43, 35.41 ppm.

FTIR-ATR: $\bar{\nu}$ = 3661 (OH), 3149 (OH), 2972 (CH), 2882 (CH), 1587 (CC aromatic), 1553 (NH), 1160 (C-O), 1038 (C-O), 843 (CH aromatic), 756 (CH aromatic), 591 (CH aromatic), 572 (CH aromatic), 436 (CC aliphatic) cm^{-1}.

Elemental analysis $C_4H_8N_2O_4S{\cdot}1H_2O$: expected C 24.24%, H 5.09%, N 14.13%; found C 24.18%, H 4.92%, N 13.39%.

Tetramethylguanidinium mesylate: [TMGH][MeSO$_3$]

The synthesis of this PIL was performed according to a previously reported method.

Tetramethylguanidinium hydrogen sulfate: [TMGH][HSO_4]

2.1 mL of tetramethylguanidine (0.01736 mol) and 1 equivalent of sulfuric acid (0.9 mL) were added to a 50 mL round-bottom flask. A total of 25 mL of acetonitrile was added and the mixture was stirred for 24h at room temperature. The solvent was evaporated and the final product was dried in vacuum and obtained as a white solid (quantitative yield).

^{1}H-NMR (δ, $CDCl_3$, 400 MHz): 2.81 (s, 1H), 1.61 (s, 12H), 0.91 (s, 2H) ppm.

^{13}C-NMR (δ, D_2O, 100 MHz): 161.38, 38.83 ppm.

FTIR-ATR: $\bar{\nu}$ = 3661 (OH), 2972 (CH), 2902 (CH), 1618 (C=C-C aromatic), 1549 (NH), 1491 (C=C-C aromatic), 1147 (C-O), 1020 (C-O), 748 (CH aromatic), 678 (CH aromatic), 626 (CH aromatic) cm^{-1}.

Elemental analysis $C_5H_{15}N_3O_4S{\cdot}0.7H_2O$: expected C 26.59%, H 7.60%, N 18.61%; found C 26.56%, H 7.16%, N 19.09%.

1,8-diazabicyclo(5.4.0)undec-7-enium mesylate: [DBUH][$MeSO_3$]

1.96 mL of 1,8-diazabicyclo(5.4.0)undec-7-ene (0.01314 mol) and 1 equivalent of methanesulfonic acid (0.85 mL) were added to a 50 mL round-bottom flask. 20 mL of acetonitrile were added and the mixture was stirred for 24h at room temperature. The solvent was evaporated and the final product was dried in vacuum and obtained as a pale yellow viscous liquid (quantitative yield).

^{1}H-NMR (δ, D_2O, 400 MHz): 3.56-3.49 (m, 4H), 3.31-3.28 (m, 2H), 2.78 (s, 3H), 2.61-2.59 (m, 2H), 2.02-1.96 (m, 2H), 1.71-1.66 (m, 6H) ppm.

^{13}C-NMR (δ, D_2O, 100 MHz): 165.95, 54.13, 48.20, 38.46, 37.96, 32.79, 28.42, 25.85, 23.30, 18.90 ppm.

FTIR-ATR: $\bar{\nu}$ = 3248 (OH), 3131 (OH), 2931 (CH), 1647 (CC aromatic), 1325 (C-N aromatic), 1156 (C-SO_2), 1038 (C-O), 771 (CH aromatic), 551 (CH aromatic), 522 (CH aromatic) cm^{-1}.

Elemental analysis $C_{10}H_{20}N_2O_3S$: expected C 48.36%, H 8.12%, N 11.28%; found C 47.98%, H 8.88%, N 11.19%.

1,8-diazabicyclo(5.4.0)undec-7-enium hydrogen sulfate: [DBUH][HSO_4]

The synthesis of this PIL was performed according to a previously reported method.

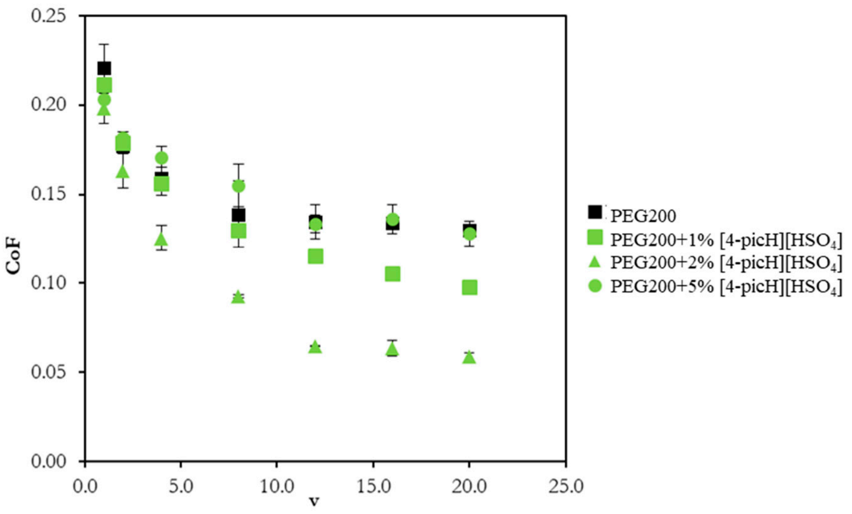

Figure S1. CoF *vs.* sliding velocity obtained with three concentrations of [4-PicH][HSO4] in PEG200: 0% (■), 1% (■), 2% (▲) and 5% (●).The errors are ± standard deviation (n≥3).

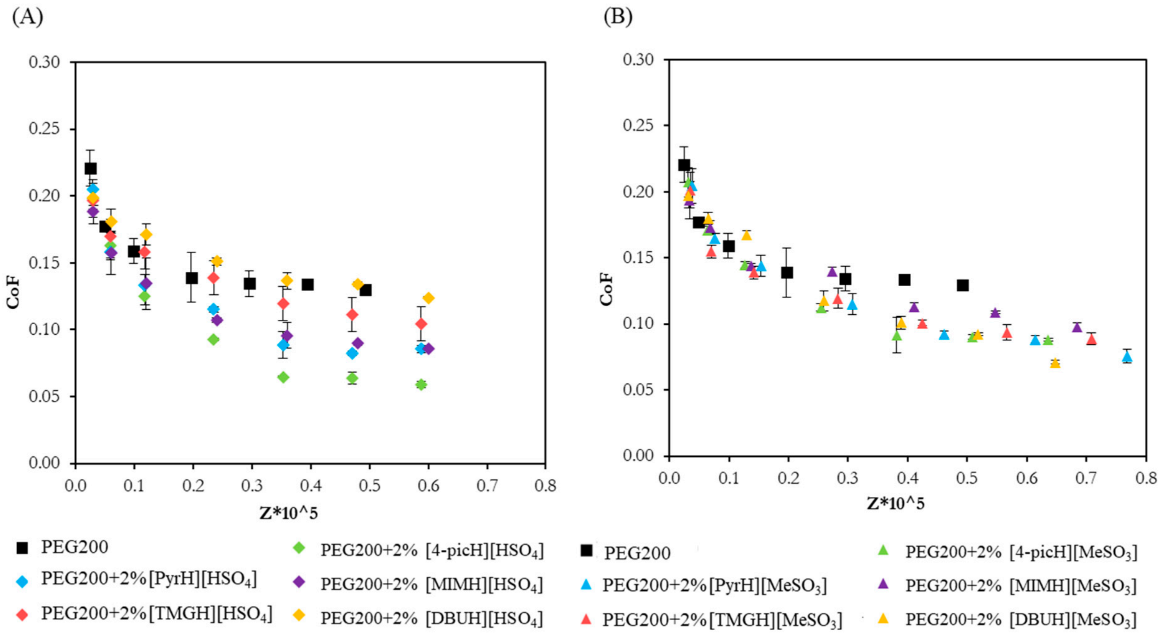

Figure S2. CoF *vs*. Sommerfeld parameter, *Z*, for neat PEG200 and the mixtures PEG200+2% PIL: (A) PILs based on the anion $[HSO_4]^-$ and (B) PILs based on the anion $[MeSO_3]^-$. The errors are ± standard deviation (n≥3).

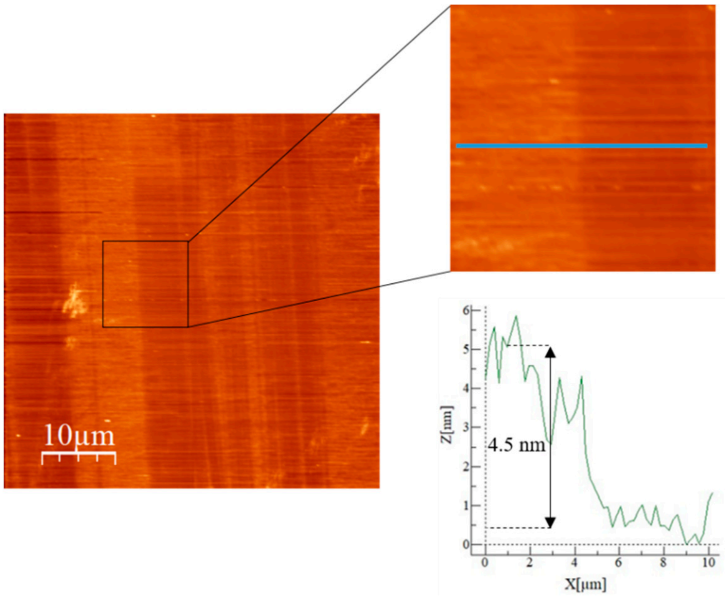

Figure S3. AFM image of the film remaining on the Si substrate after the tribological test with PEG200+2% [4-picH][HSO_4] (2375 cycles, 1N) . The insert represents the magnification of marked area on the track an adsorbed layer.

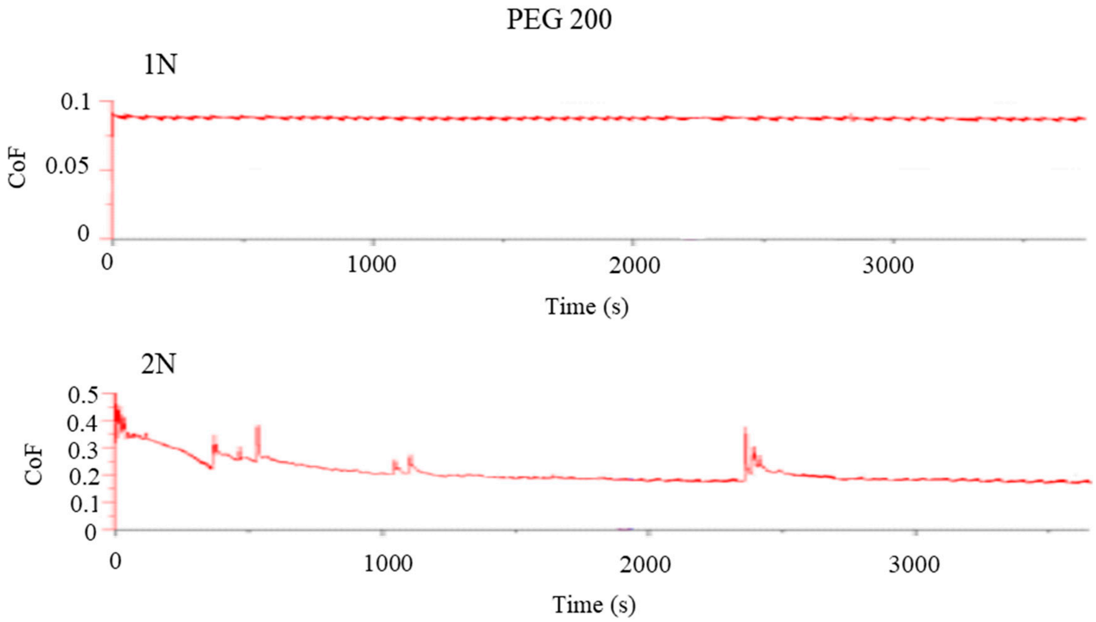

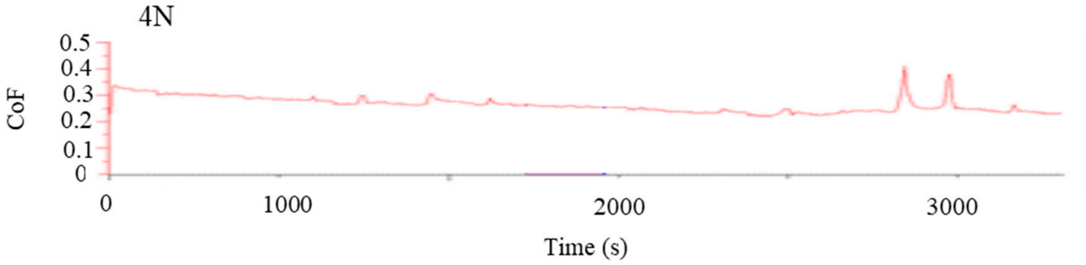

PEG200+2% [4-picH][$MeSO_3$]

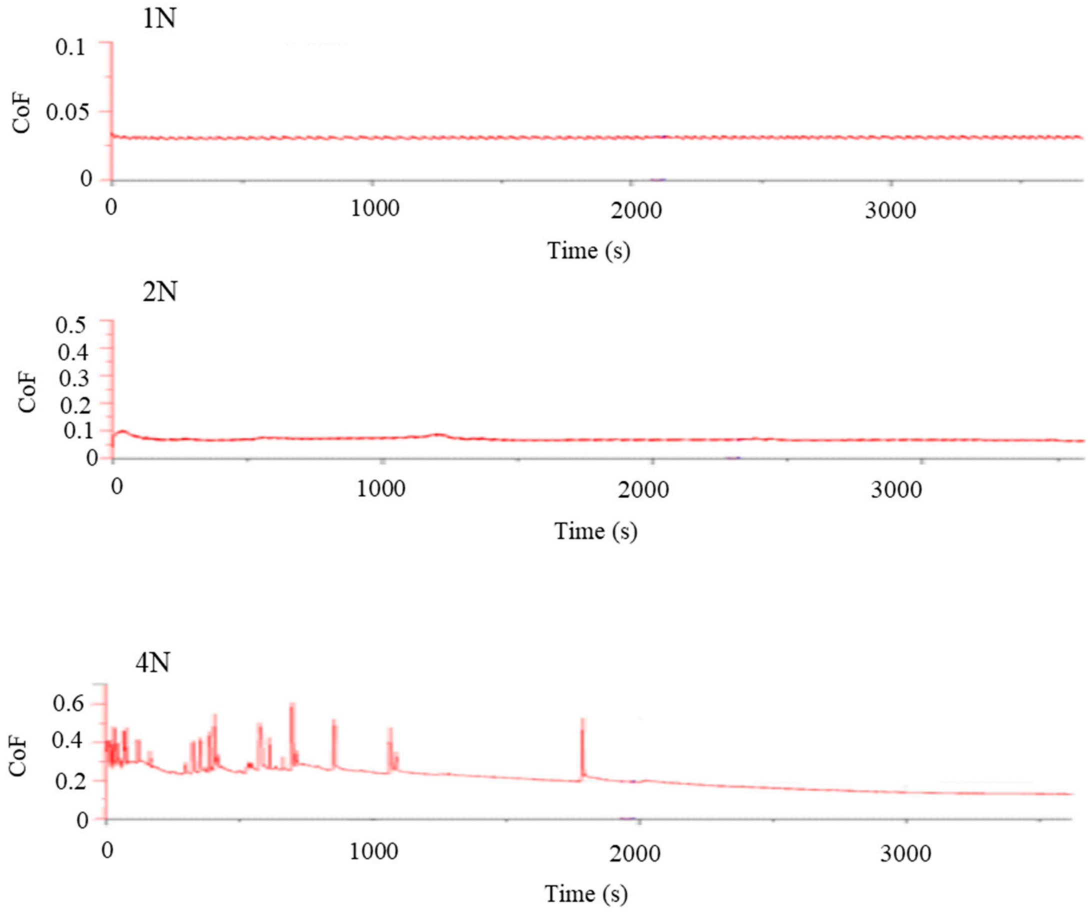

PEG200+2% [4-picH][HSO_4]

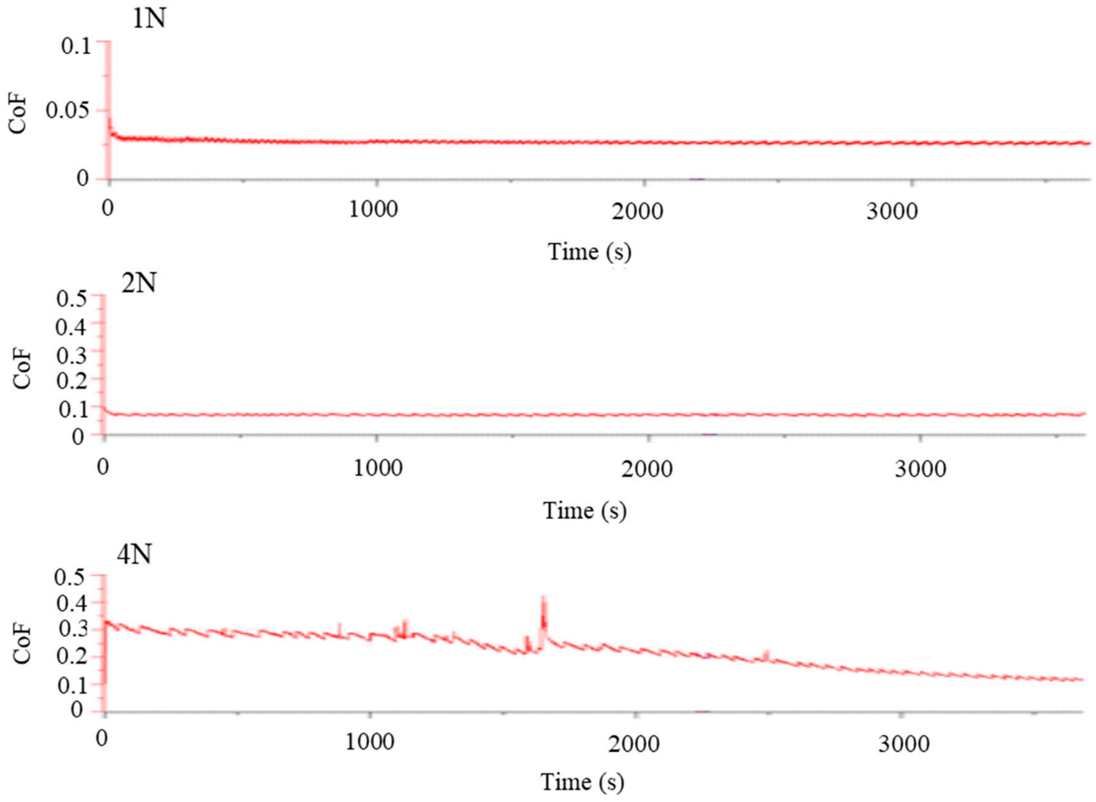

PEG200+2% [DBUH][$MeSO_3$]

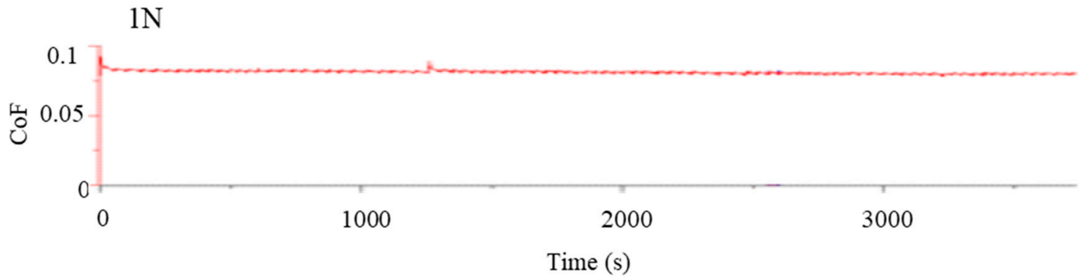

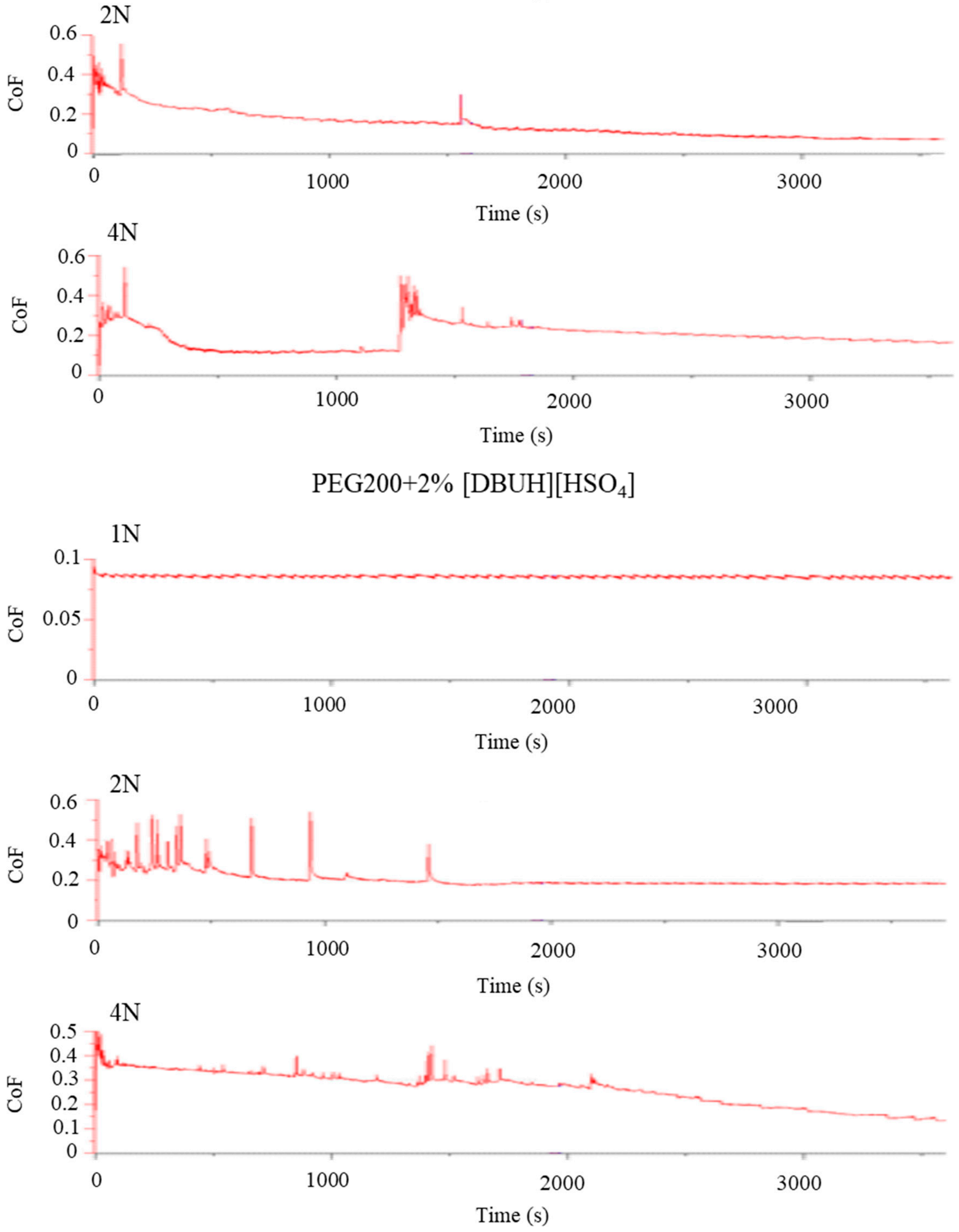

Figure S4. CoF as a function of time obtained in long tests with the pair Si/Si, under 1N, 2N and 4N, and v=8mm·s^{-1} using PEG200 and the mixtures of PEG200 with [4-picH][MeSO$_3$], [4-picH][HSO$_4$], [DBUH][MeSO$_3$] and [DBUH][HSO$_4$] as lubricants.

[4-picH][MeSO3]

^{1}H NMR

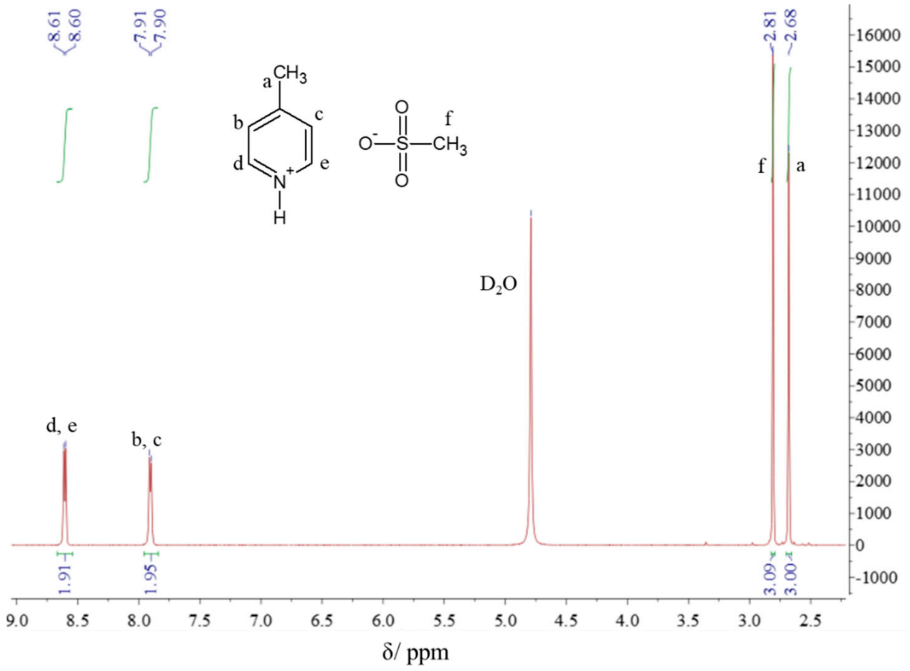

^{13}C NMR

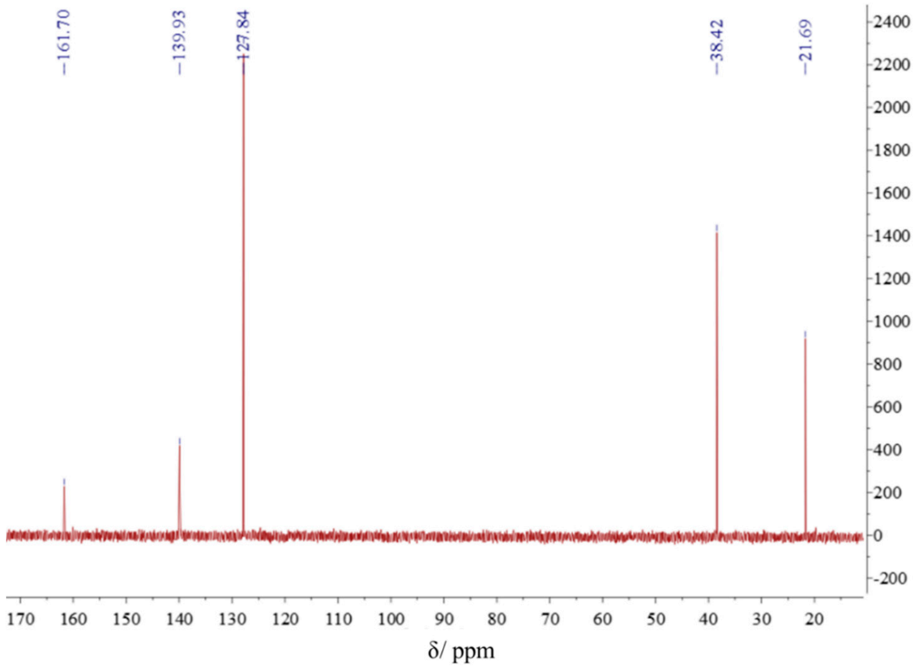

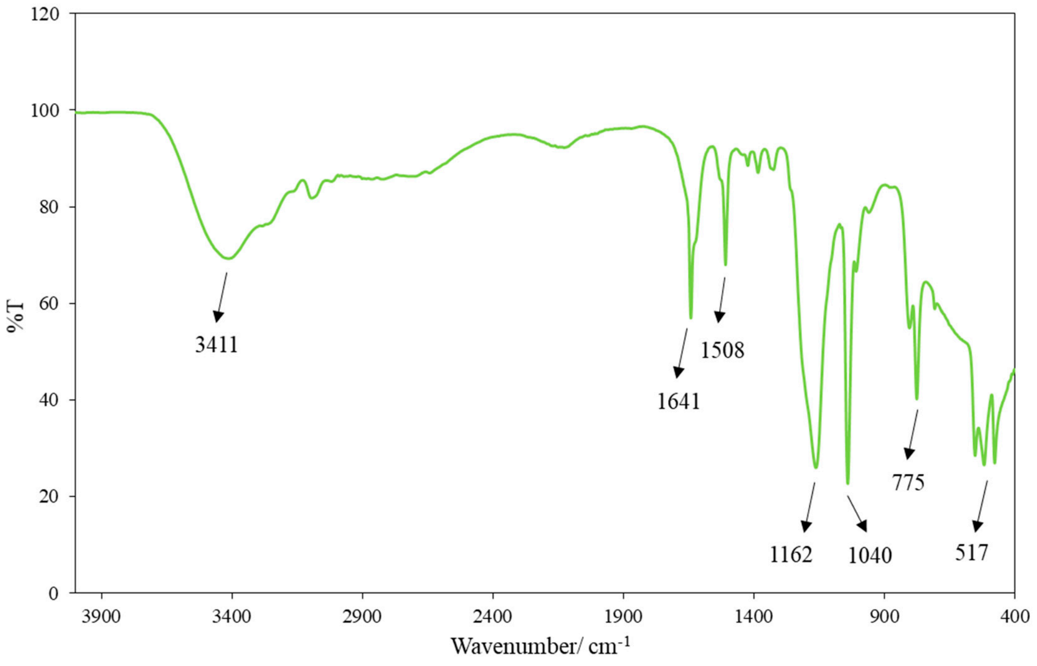

[4-picH][HSO$_4$]

^{1}H NMR

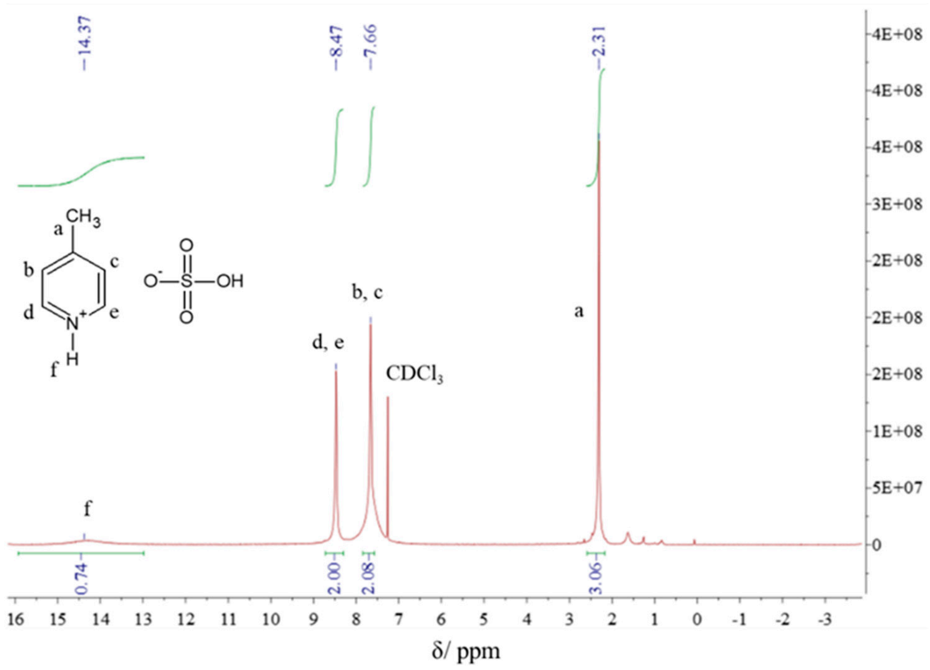

^{13}C NMR

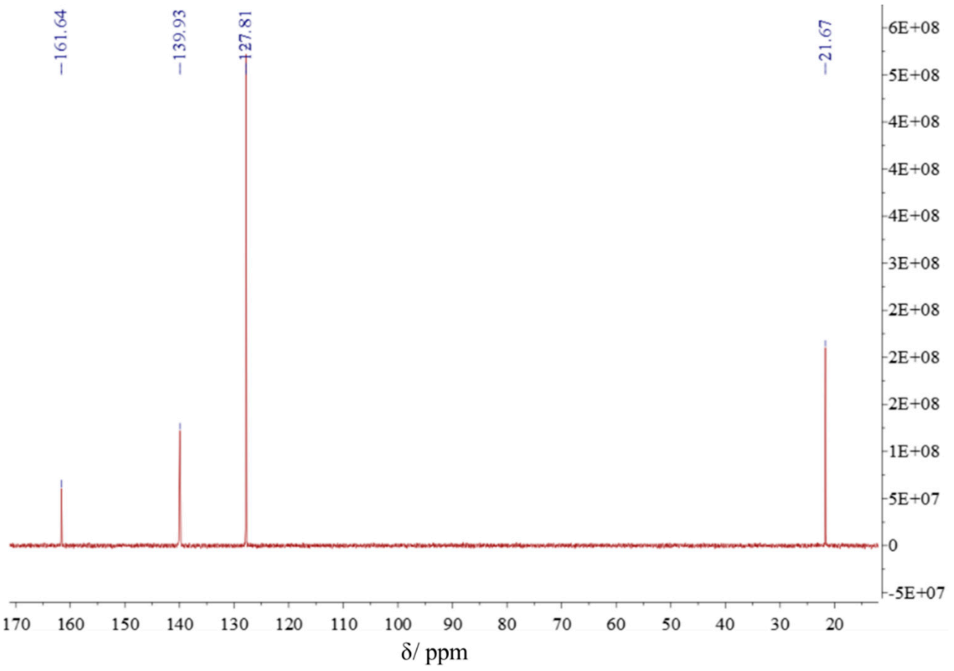

161.64
139.93
127.81
21.67
6E+08
5E+08
4E+08
4E+08
4E+08
3E+08
2E+08
2E+08
2E+08
1E+08
5E+07
0
-5E+07
170 160 150 140 130 120 110 100 90 80 70 60 50 40 30 20
δ/ ppm

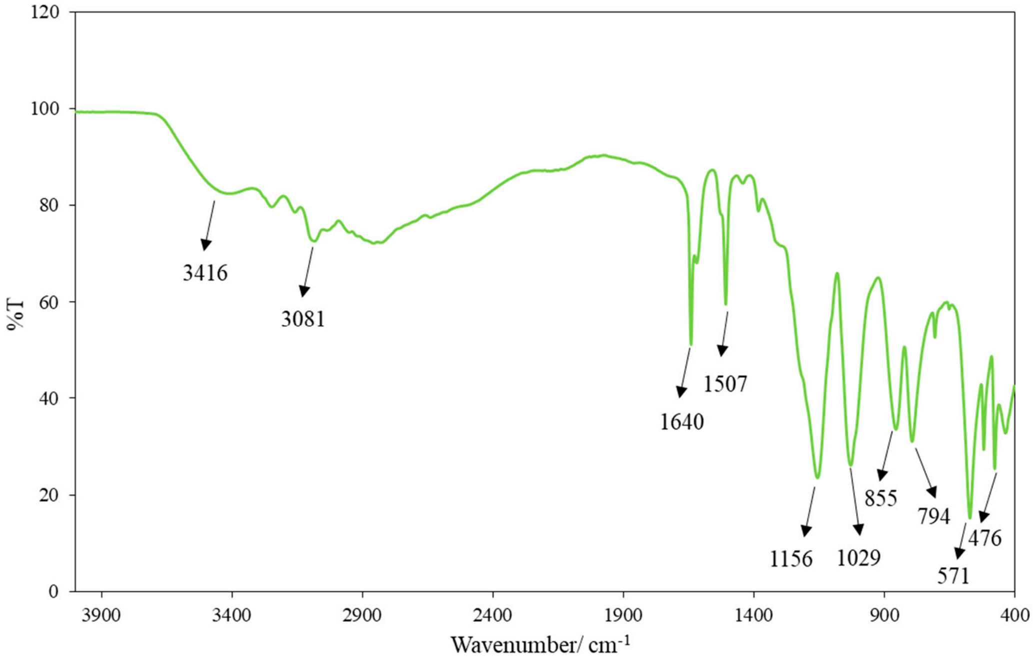

120
100
80
60
40
20
0
%T
3416
3081
1640
1507
1156
1029
855
794
571
476
3900 3400 2900 2400 1900 1400 900 400
Wavenumber/ cm-1

[PyrH][MeSO3]

^{1}H NMR

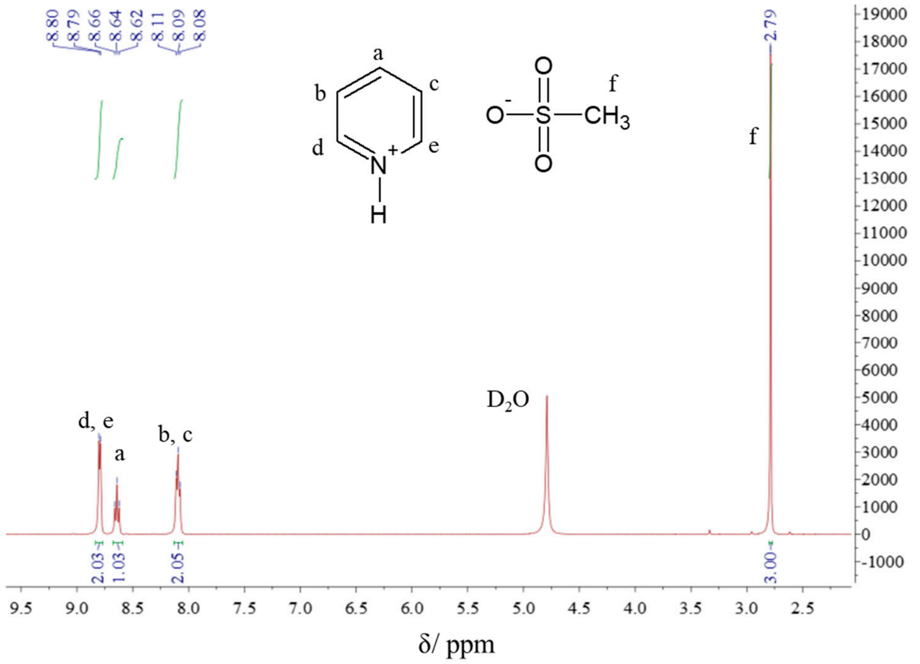

^{13}C NMR

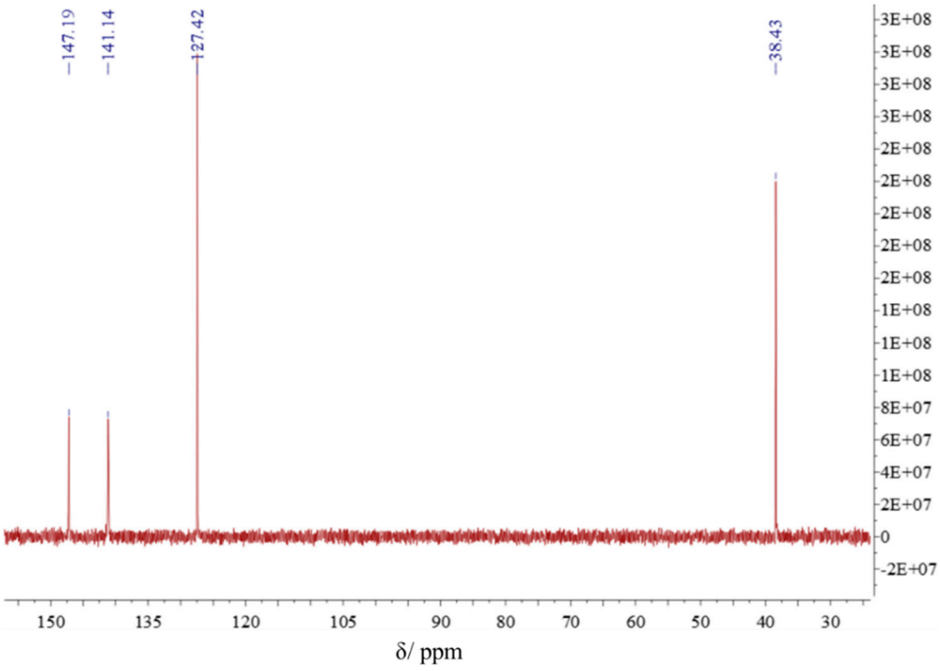

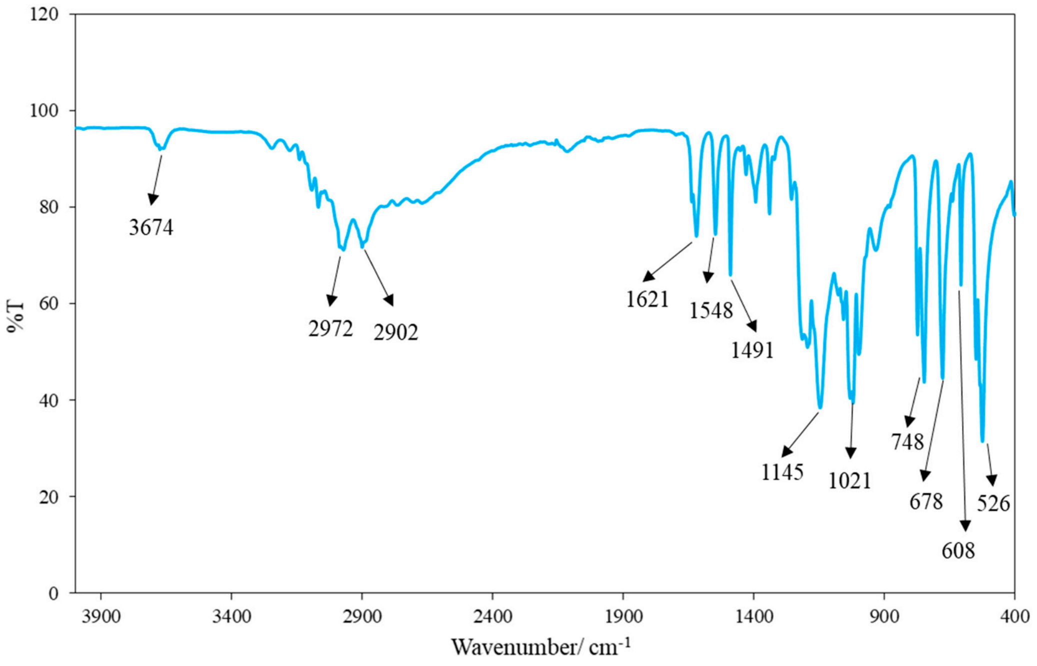

[PyrH][HSO_4]

^{1}H NMR

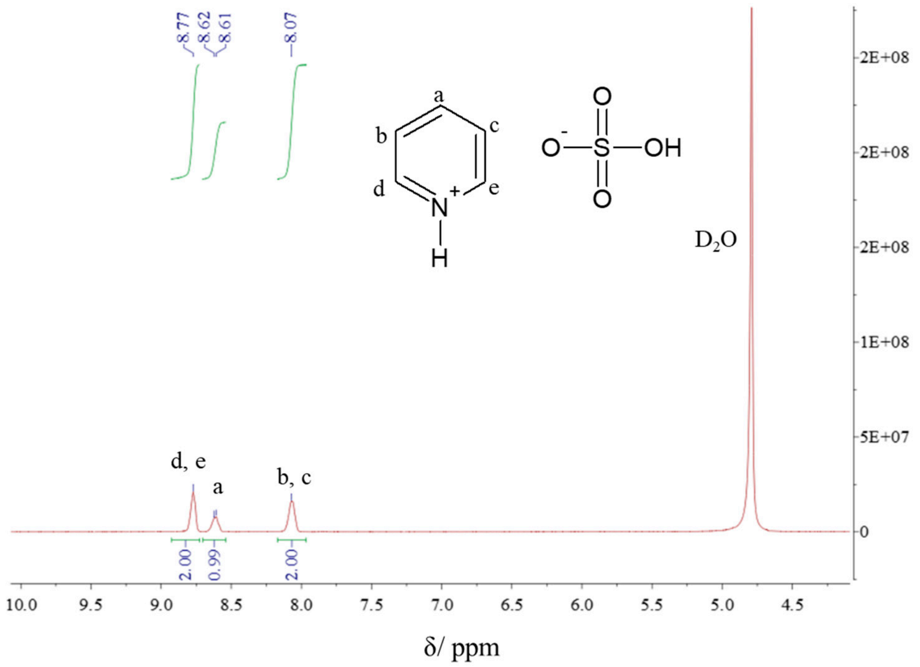

^{13}C NMR

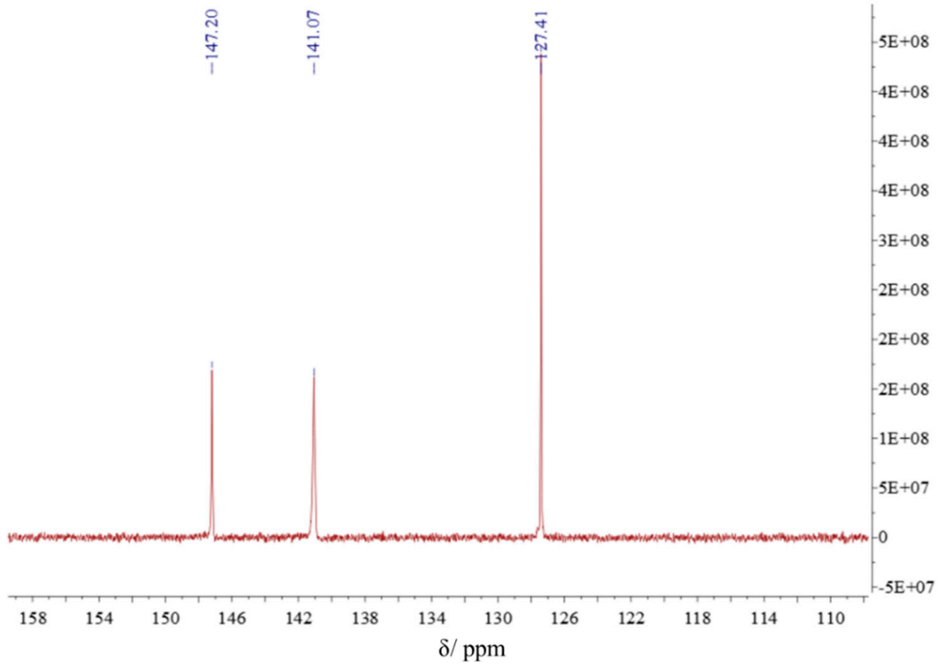

147.20
141.07
127.41
5E+08
4E+08
4E+08
4E+08
3E+08
2E+08
2E+08
2E+08
1E+08
5E+07
0
-5E+07
158
154
150
146
142
138
134
130
126
122
118
114
110
δ/ ppm

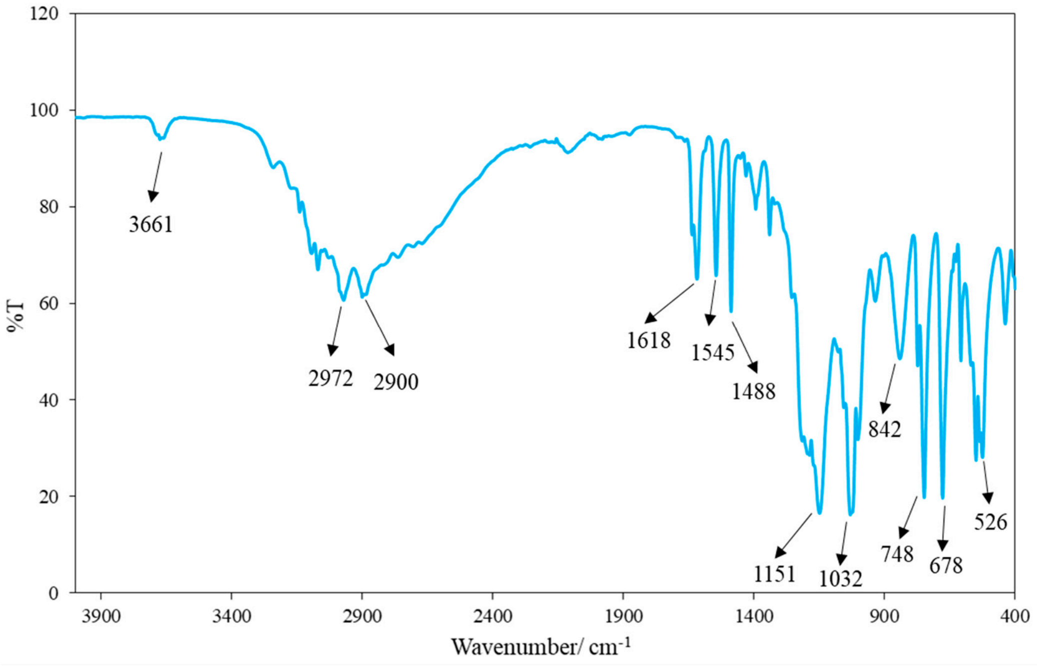

120
100
80
60
40
20
0
%T
3661
2972
2900
1618
1545
1488
842
1151
1032
748
678
526
3900
3400
2900
2400
1900
1400
900
400
Wavenumber/ cm-1

[MIMH][HSO_4]

^{1}H NMR

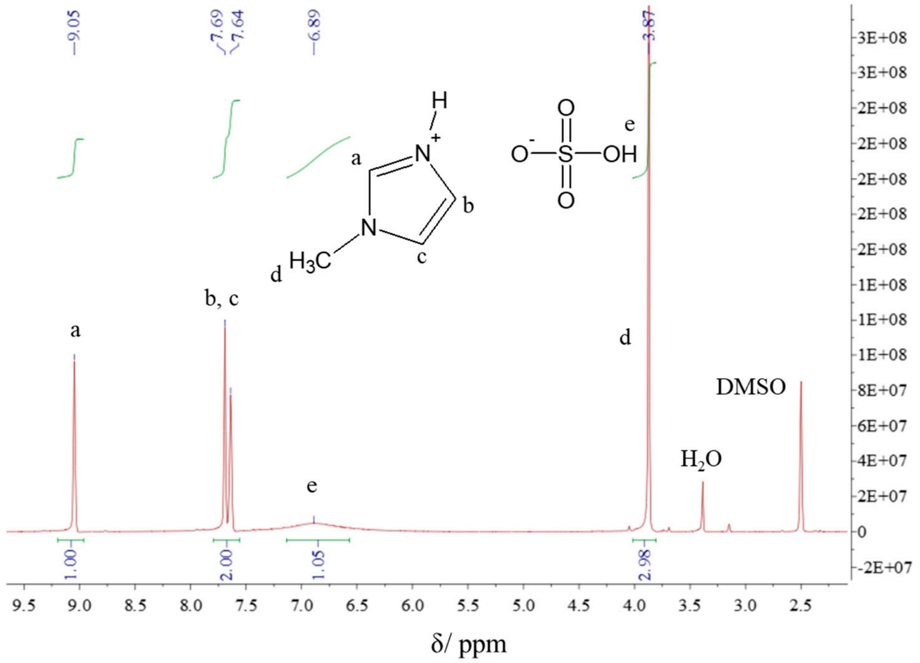

^{13}C NMR

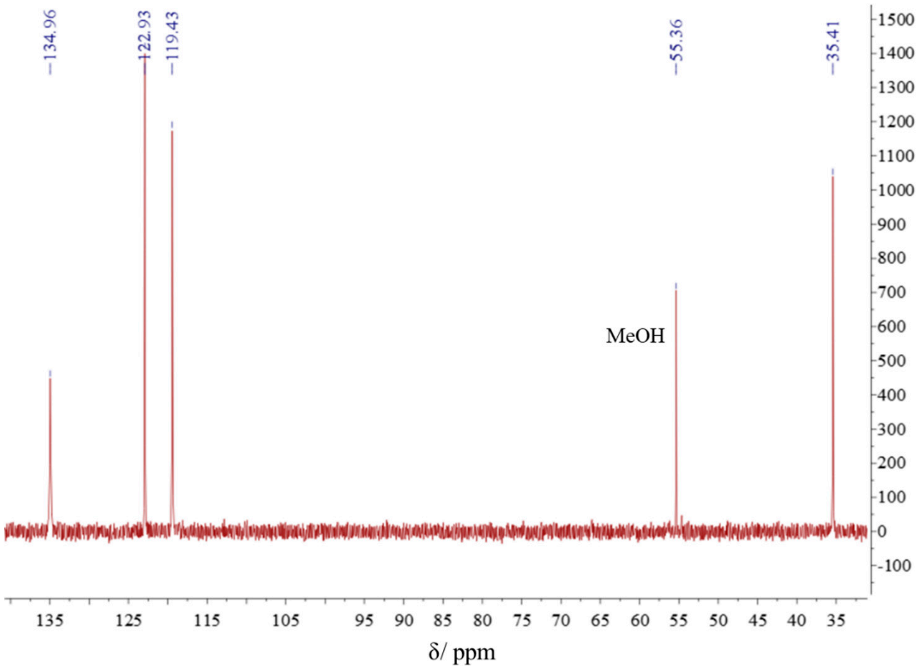

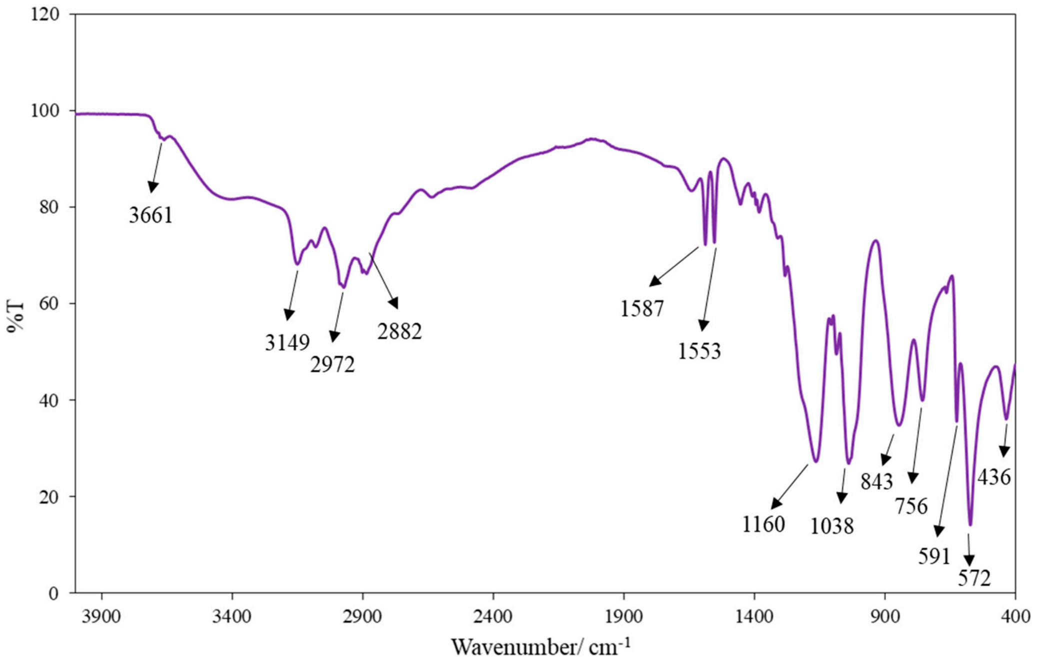

[TMGH][HSO_4]

^{1}H NMR

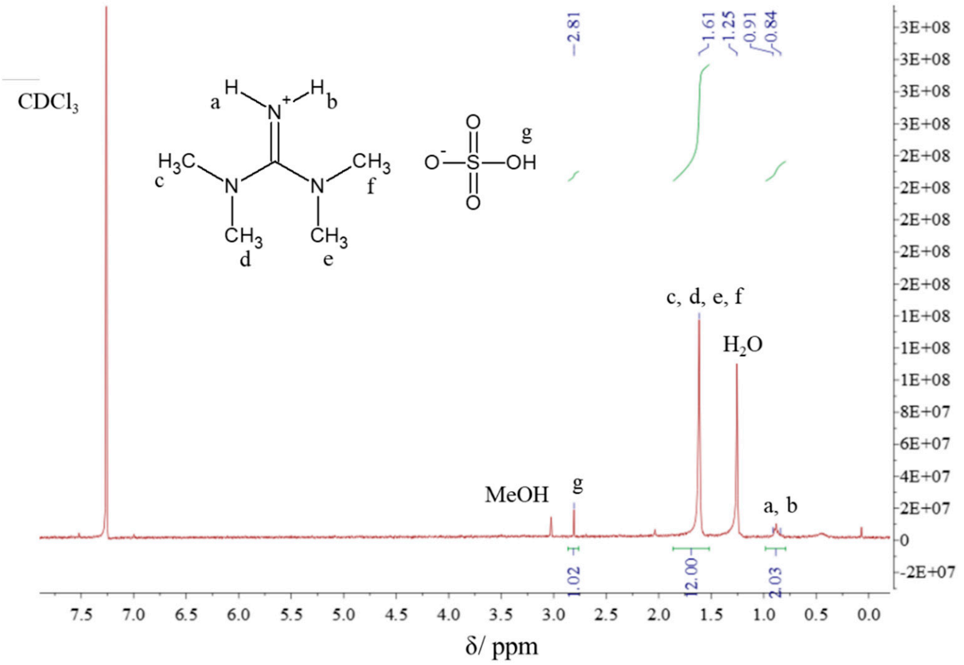

^{13}C NMR

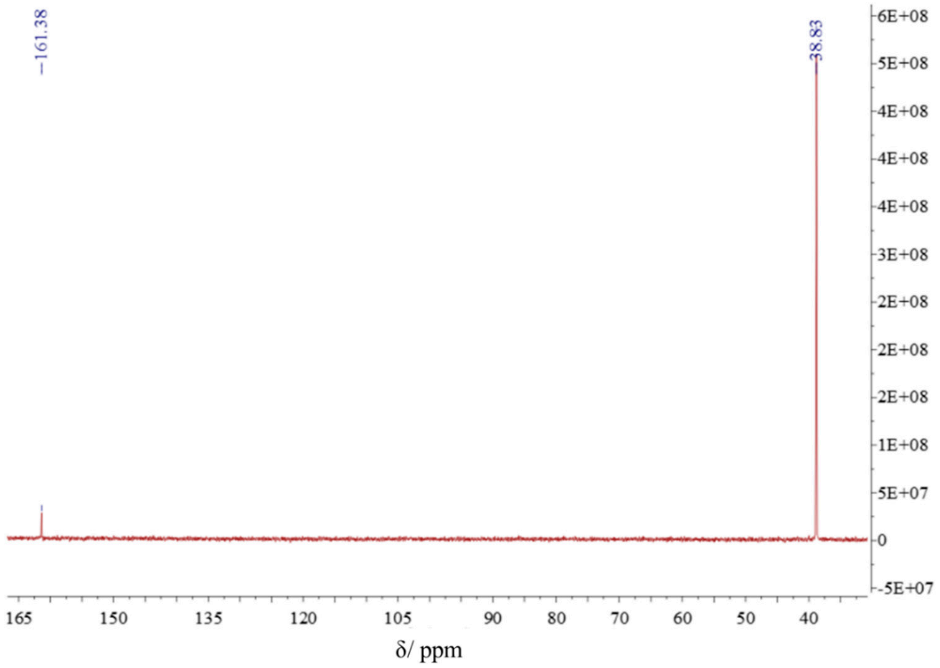

161.38
38.83
6E+08
5E+08
4E+08
4E+08
4E+08
3E+08
2E+08
2E+08
2E+08
1E+08
5E+07
0
-5E+07
165
150
135
120
105
90
80
70
60
50
40
δ/ ppm

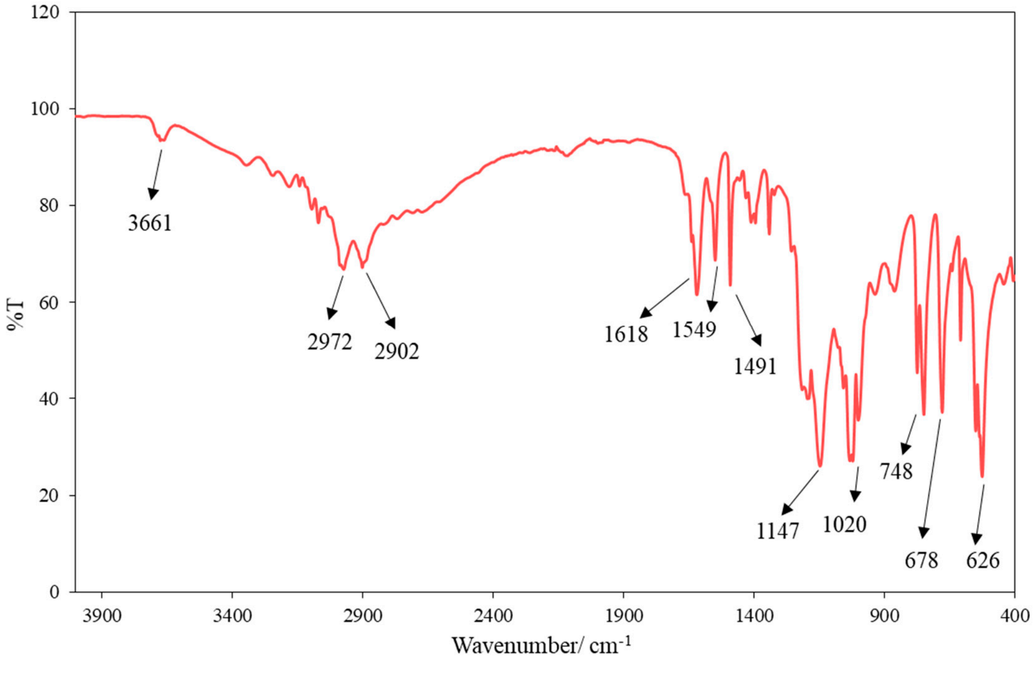

120
100
80
60
40
20
0
%T
3661
2972
2902
1618
1549
1491
1147
1020
748
678
626
3900
3400
2900
2400
1900
1400
900
400
Wavenumber/ cm-1

[DBUH][MeSO3]

^{1}H NMR

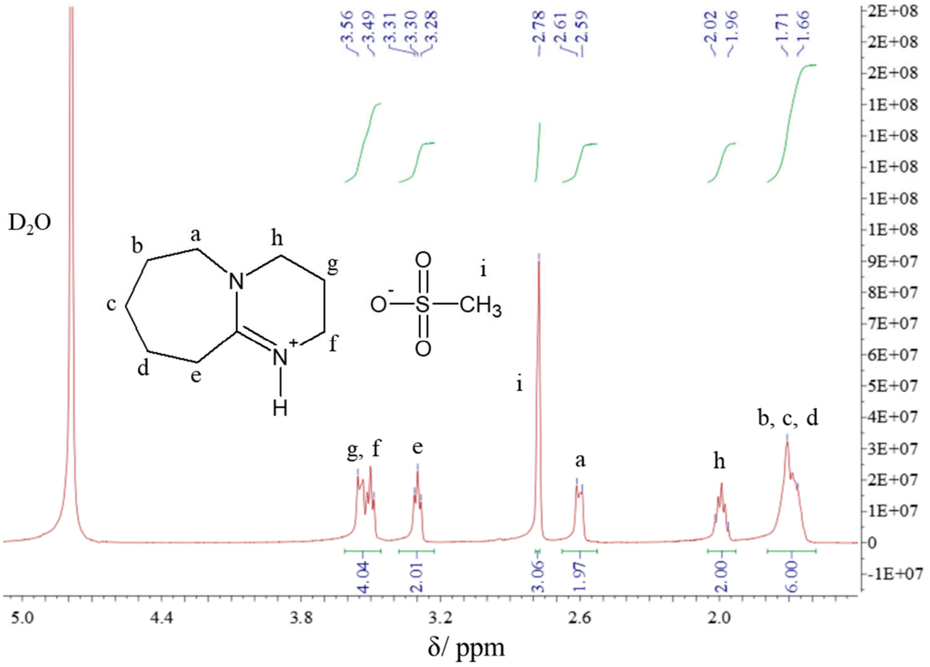

^{13}C NMR

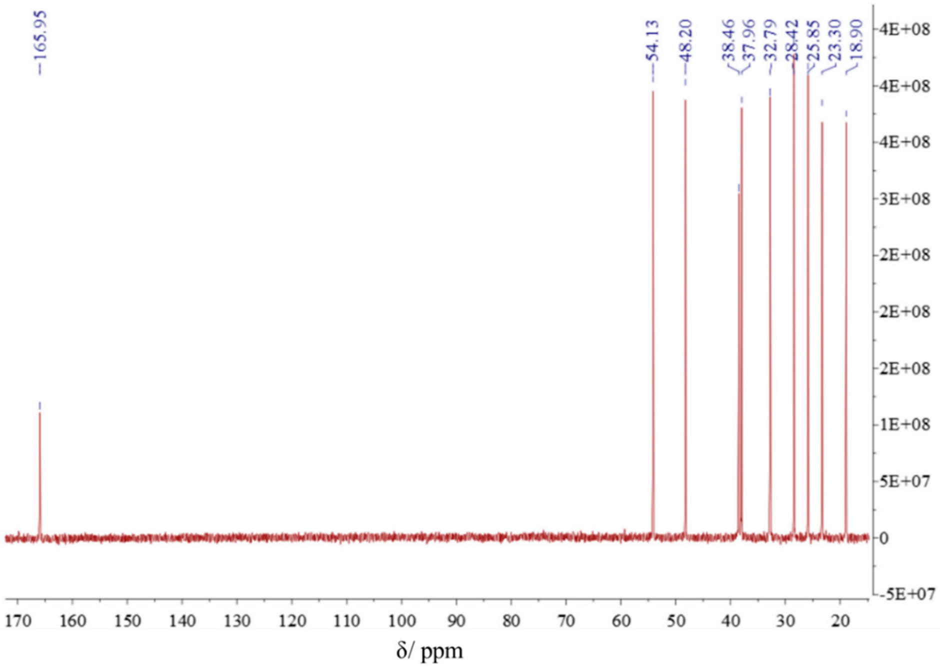

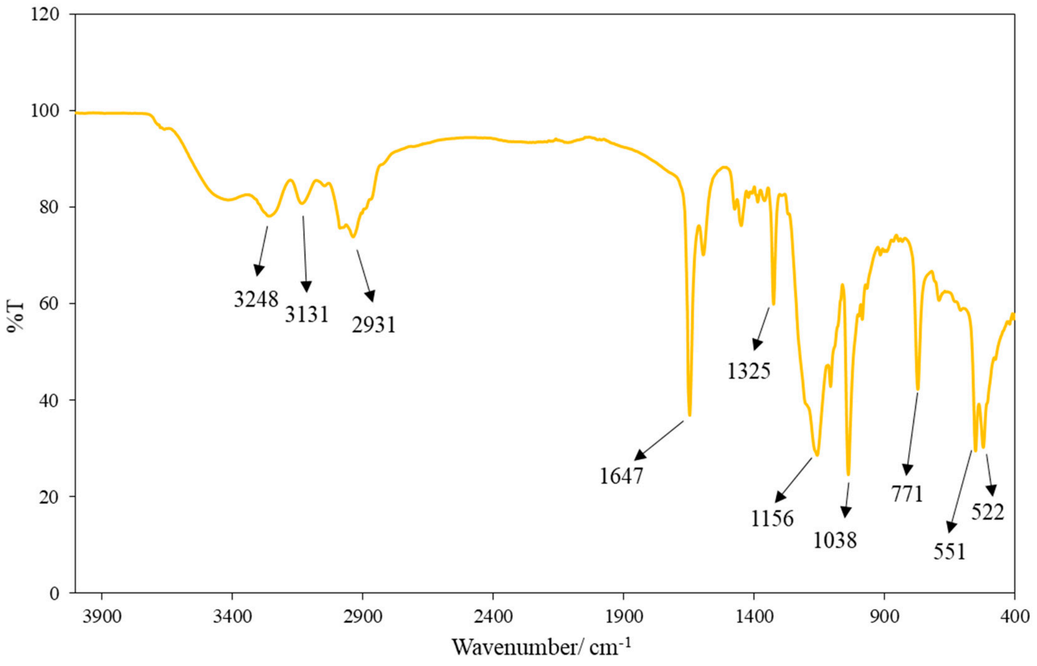

Figure S5. ^{1}H-NMR, ^{13}C-NMR and FTIR spectra of the synthesized PILs.

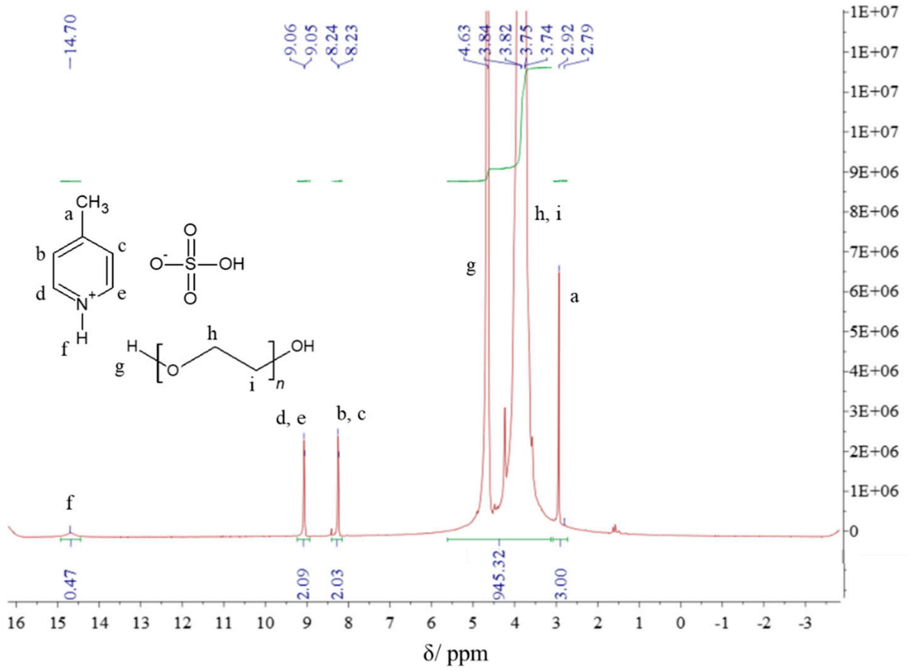

Figure S6. ^{1}H-NMR spectrum of the mixture PEG200+2% [4-picH][HSO_4].

Calculation of the theoretical minimum film thickness using elastohydrodynamic theory of lubrication (EHL)

According to the elastohydrodynamic theory of lubrication (EHL) applied to non-conformal geometry of ball-on-disk contact, the theoretical minimum film thickness, h, depends on the viscosity at atmospheric pressure (η_{atm}), and pressure-viscosity coefficient (α) of the lubricant and reduced modulus of the contact between the surfaces (E_r). The value of E_r was calculated using Equation (S1):

$$\frac{1}{E_r} = \frac{1}{2}\left[\left(\frac{1-\vartheta_{disk}^2}{E_{disk}}\right) + \left(\frac{1-\vartheta_{ball}^2}{E_{ball}}\right)\right] \quad \text{(S1)}$$

where the values of Poisson's ratio and Young's modulus for Si, taken from reference [1], are 0.27-0.22 and 130-165 GPa, respectively.

The theoretical minimum film thickness, h, can be calculated using the Hamrock model [2], through the following equation:

$$h = 3.63R\left(\frac{U\eta_{atm}}{E_r R}\right)^{0.68} (\alpha E_r)^{0.49} \left(\frac{W}{E_r R^2}\right)^{-0.073} (1 - e^{-0.68k}) \quad (2)$$

where R is half of the radius of the ball, U is half of the sliding speed (as an approximation, in reciprocating movement, can be taken as the average sliding speed of the test), W is the load and k is ellipticity parameter (taken as 1 for point contact). The pressure-viscosity coefficient, $\alpha = \frac{1}{\eta}\left(\frac{\partial \eta}{\partial P}\right)_T$, was calculated as 17 GPa^{-1} for tetraethylene glycol, from data reported in the literature at 25 ºC.[3] The same value was used, as an approximation, for the PEG200+[4-picH][HSO_4] mixture, since PEG200 and tetraethylene glycol have very similar molecular weights and the solution of IL in PEG200 is very diluted.

References

[1] Hopcroft, M. A.; Nix, W. D.; Kenny, T. W. What Is the Young's Modulus of Silicon? *J. Microelectromechanical Syst.* **2010**, *19* (2), 229–238. https://doi.org/10.1109/JMEMS.2009.2039697

[2] Hamrock, B. J.; Dowson, D. Isothermal Elastohydrodynamic Lubrication of Point Contacts: Part III—Fully Flooded Results**.** *J. Lubr. Technol.,* **1977**, 99, 264-275. https://doi.org/10.1115/1.3453074

[3] Pereira, M. F. V.; Avelino, H. M. N. T.; Caetano, F. J. P.; Fareleira, J. M. N. A. Viscosity of Liquid Diethylene, Triethylene and Tetraethylene Glycols at Moderately High Pressures Using a Vibrating Wire Instrument. *Fluid Phase Equilibria* **2019**, *480*, 87–97. https://doi.org/10.1016/j.fluid.2018.09.026
